# Diagnostic and management practices for phenylketonuria in 19 countries of the South and Eastern European Region: survey results

**DOI:** 10.1007/s00431-015-2622-5

**Published:** 2015-09-08

**Authors:** Maria Giżewska, Anita MacDonald, Amaya Bélanger-Quintana, Alberto Burlina, Maureen Cleary, Turgay Coşkun, François Feillet, Ania C. Muntau, Friedrich K. Trefz, Francjan J. van Spronsen, Nenad Blau

**Affiliations:** Department of Pediatrics, Endocrinology, Diabetology, Metabolic Diseases and Cardiology, Pomeranian Medical University, 71-252 Szczecin; Unii Lubelskiej 1, Szczecin, Poland; Department of Dietetics, The Children’s Hospital, Birmingham, UK; Hospital Ramon y Cajal, Madrid, Spain; Department of Pediatrics, University Hospital, Padova, Italy; Department of Metabolic Medicine, Great Ormond Street Hospital, London, UK; Department of Pediatrics, Division of Pediatric Nutrition and Metabolism, Hacettepe University Faculty of Medicine, Hacettepe, Ankara, Turkey; Hôpital d’Enfants, CHU Brabois, Vandoeuvre-lès-Nancy, France; University Children’s Hospital, University Medical Center Hamburg Eppendorf, Hamburg, Germany; University Children’s Hospital, Heidelberg, Germany; Department of Paediatrics, University of Groningen, Groningen, The Netherlands; Dietmar-Hopp Metabolic Center, University Children’s Hospital, Heidelberg, Germany

**Keywords:** Diagnosis, Management, Phenylketonuria, Questionnaire, Sapropterin dihydrochloride, Screening, Survey, Tetrahydrobiopterin

## Abstract

To avoid potentially severe outcomes, phenylketonuria (PKU) must be detected as soon as possible after birth and managed with life-long treatment. A questionnaire-based survey was performed to document diagnosis and management practices for PKU in a region of Southern and Eastern Europe. Prevalence and management data were obtained from 37/59 (63 %) centres within 19/22 (86 %) contacted countries (*N* = 8600 patients). The main results’ analysis was based on completed questionnaires obtained from 31 centres (53 %) within 15 countries (68 %). A median of 10 % of patients per centre had been diagnosed after the newborn period. Metabolic dieticians and specialised adult PKU clinics were lacking in 36 and 84 % of centres, respectively. In 26 % of centres, treatment initiation was delayed until >15 days of life. Blood phenylalanine (Phe) thresholds to start treatment and upper Phe targets were inconsistent across centres. Ten percent of centres reported monitoring Phe every 2 weeks for pregnant women with PKU, which is insufficient to minimise risk of neonatal sequalae. Sapropterin dihydrochloride treatment was available in 48 % of centres, with 24-h responsiveness tests most common (36 %). Only one centre among the five countries lacking newborn screening provided a completed questionnaire.

*Conclusion*: Targeted efforts by health care professionals and governments are needed to optimise diagnostic and management approaches for PKU in Southern and Eastern Europe.

**Table:** 

“**What is Known**” • *PKU must be detected early and optimally managed throughout life to avoid poor outcomes, yet newborn screening is not universal and diagnostic and management practices for PKU are known to vary widely between different centres and countries.* • *Targeted efforts by health care professionals and governments are needed to optimise diagnostic and management approaches.*
“**What is New**” • *PKU management practices are documented in 19 South and Eastern European countries indicating a heterogeneous situation across the region.* • *Key areas for improvement identified in surveyed centres include a need for comprehensive screening in all countries, increased number of metabolic dietitians and specialised adult PKU clinics, delayed time to treatment initiation, appropriate Phe thresholds, Phe targets and monitoring frequencies, and universal access to currently available treatment options.*

**Electronic supplementary material:**

The online version of this article (doi:10.1007/s00431-015-2622-5) contains supplementary material, which is available to authorized users.

## Introduction

Phenylketonuria (PKU; OMIM: #261600) is an inborn error of phenylalanine (Phe) metabolism with an estimated average prevalence in Europe of 1/10,000 live births [16]. Timely newborn screening and life-long Phe-restricted diet enable the severe outcomes of untreated PKU to be avoided [2]. Despite the cost-effective nature of newborn screening, it has been reported that several countries either do not offer this service or can offer it to only part of their population [3–5, 9, 10, 15, 20, 22–24, 30]. Furthermore, diagnostic and management practices for PKU vary between countries. The present study was conducted to describe the management and treatment practices for PKU in an area comprising much of South and Eastern Europe. The majority of these centres were not captured by a previous European survey [1]. Based on socioeconomic factors such as gross domestic product per capita, some of these countries might be expected to employ less comprehensive management approaches for PKU than are in use in more affluent regions of Western Europe. This survey was designed to highlight the shortcomings in PKU management and to facilitate the targeting of future initiatives.

## Methods

### Questionnaire development

A questionnaire consisting of 52 closed (answer choices provided) and 29 open answer questions was developed covering the following topics: (1) general information; (2) screening procedures and confirmatory diagnosis procedures; (3) treatment practices; (4) follow-up; (5) constitution of treatment team; (6) existing guidelines and protocols; (7) services offered to patients; and (8) challenges and areas for improvement in PKU management. The full survey is available in Online Resource 1.

### Invited health care professionals

**Fig. 1:**
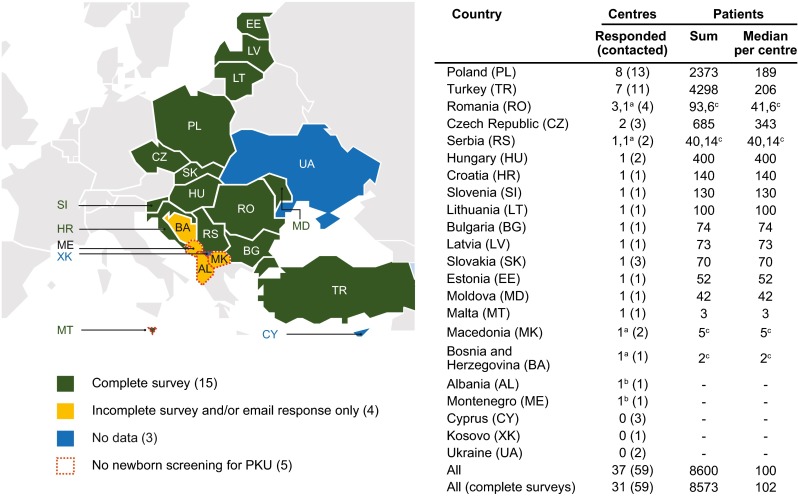
Countries and centres participating in the survey. Information was returned from 19/22 contacted countries, ordered in the list by the total number of responding centres per country (high to low). ‘–’ data not provided. ^a^Centre provided only an incomplete questionnaire response; ^b^Centre provided only an email response with limited information; ^c^Number of patients in centre providing the incomplete questionnaire, not included in main results’ analysis

An invitation with a link to the online questionnaire (Survey Monkey: www.surveymonkey.com) was emailed to 80 health care professionals (HCP) working in the field of PKU from 59 centres in 22 countries in South and Eastern Europe (see Fig. 1 and Appendix). Belarus was not included in this study because no HCP contacts were known. The Russian Federation was not included due to difficulties in accessing health professionals, although many PKU treatment centres exist. The questionnaire was only provided in English.

Data were collected from February to August 2014. A maximum of three HCPs per centre were invited to participate. To avoid inconsistencies in responses, all contributing HCPs from the same centre were requested to complete a single questionnaire.

### Data analysis

Returned questionnaires were analysed for completeness: questionnaires with incomplete answers beyond the ‘general information’ section were excluded from the main results analysis, and the demographic data provided by these questionnaires were evaluated separately (see Sect. “Centres providing demographic data only/e-mail responses”). Information provided by centres exclusively in e-mail form was also evaluated separately (see Sect. “Centres providing demographic data only/e-mail responses”).

Data were analysed using descriptive statistics (percent of total responses or medians). Prior to analysis, responses to some open answer questions were grouped or categorised according to the answers received. Answers were not systematically quality checked with the participating centres. Questions and answers not included in this report are summarised in Online Resource 2.

## Results

### Contributing centres and HCPs

In total, information on the prevalence and management of PKU was obtained from 37/59 (63 %) centres in 19/22 (86 %) contacted countries (Fig. 1). Complete questionnaires were returned from 31 centres involving 60 HCPs in 15 countries. Incomplete questionnaires, which included only demographic data, and/or e-mail-only responses were returned from six centres involving an additional four countries (Fig. 1; see Sect. “Centres providing demographic data only/e-mail responses” for more details). Contributing HCPs and centres are listed in Appendix.

Among the 60 HCPs returning complete questionnaires, most were either metabolic paediatricians (43.3 %) or paediatricians (30.0 %). Other professions included ‘clinical geneticist’ (6.7 %); ‘dietician/nutritionist’ (6.7 %); ‘research scientist’ (3.3 %); and ‘dietician, metabolic diseases’, ‘laboratory technician’, ‘physician’, ‘adult metabolic doctor’, ‘nurse specialising in PKU’ and ‘clinical biochemist’ (each 1.7 %). These HCPs had a median (range) of 17 (1–41) years of PKU clinical experience, and 75 % cared for patients of all ages with PKU or maternal PKU.

Ten percent of centres reported that their PKU diagnosis and treatment team consisted of physicians, clinical biochemists, psychologists, dieticians and specialist nurses who care for the PKU population, and 16.1 % of centres reported having a specialised adult PKU clinic (Table 1). A dedicated dietician was part of the team in 65 % of centres, 70 % of whom have a university degree.

**Table 1:** Summary of patients with PKU and management team profile (*N* = 31)

Question and answers by category							Median (range)
How many PKU patients are currently followed at your clinic/centre?							102 (3–2500)
1–25	26–50	51–100	101–200	201–500	≥501		
12.9 %	9.7 %	22.6 %	16.1 %	22.6 %	12.9 %		
What is the number of new PKU patients followed over the course of a typical year at your clinic/centre?							6 (1–50)
0	1–5	6–10	11–20	≥21	No answer		
0 %	46.7 %	20.0 %	13.3 %	13.3 %	6.7 %		
What is the number of maternal PKU pregnancies followed over the course of a typical year at your clinic/centre?							2 (0–17)
0	1–5	6–10	11–20	≥21	No answer		
19.4 %	58.1 %	6.5 %	3.2 %	0 %	12.9 %		
Approximately what percentage of PKU patients currently followed at your centre were late diagnosed?							10 (0–100)
0 %	1–5 %	6–10 %	11–20 %	≥21 %	No answer		
6.5 %	29.0 %	19.4 %	25.8 %	12.9 %	6.5 %		
The PKU team at your centre includes which of the following?^a^							N/A
Physicians (any type)	Clinical biochemists	Psychologists	Dieticians/nutritionists (any type)	Nurses specialising in PKU	Research scientists	Other^b^	
100 %	58.1 %	54.8 %	71.0 %	25.8 %	29.0 %	9.7 %	
Who cares for PKU patients from the age of 18 years?^a^							N/A
PKU paediatric clinic			PKU adult clinic		Other^c^		
71.0 %			16.1 %		16.1 %		

*N/A* not applicable, *PKU* phenylketonuria

^a^Multiple answers were possible, and therefore, the total exceeds 100 %

^b^‘Other’ was specified as ‘molecular geneticist’, ‘laboratory technician’, and ‘neurologist’ in three surveys

^c^‘Other’ was specified as (number of questionnaires): ‘Department of Genetics’ (3), ‘adult neurologists (1), ‘metabolic physicians’ (1) and ‘general practitioner’ (1)

### Patients

In total, the 31 centres who returned complete questionnaires followed a total of 8573 patients with PKU, of whom approximately 75 % were followed in two countries, Turkey and Poland (Fig. 1). Data on patients and the HCPs working in the treatment team at each centre are presented in Table 1. The median number of patients per centre was 102 (range 3–2500), with the largest centres in Turkey and Poland (Fig. 1). The median number of new patients followed per year per centre was 6 (range 1–50). A median of 10 % (range 0–100) of patients were late diagnosed, and only two centres had no late diagnosed patients. Across the region, patients were estimated to travel a median of 110 km (range 10–500 km) to their centre.

### Screening and confirmatory diagnosis procedures

Among the completed questionnaires, only one centre in Malta had no newborn screening programme (Fig. 1, further information on screening practices presented in Table 2; see Sect. “Centres providing demographic data only/e-mail responses” for responses from other countries lacking newborn screening). Seventy-one percent of centres screened at 3 days of age (median 3 days; range 2–7 days), and 77 % saw positively screened newborns within the first 15 days of life (median 10 days; range 3–30 days). The most common upper threshold for a positive newborn screening test was a blood Phe concentration of 120 μmol/L (48.4 %), followed by 180 μmol/L (25.8 %).

**Table 2:** Summary of screening and confirmatory diagnostic practices (*N* = 31)

Question and answers by category							Median (range)
At what age is the heel prick test performed at your centre?							3 (2–7)
0–1 days	2 days	3 days	4 days	≥5 days		No answer	
0 %	19.4 %	71.0 %	3.2 %	3.2 %		3.2 %	
At what age are positively screened newborns seen in the medical service?							10 (3–30)
0–9 days	10–15 days	16–28 days		≥29 days		No answer	
41.9 %	35.5 %	16.1 %		3.2 %		3.2 %	
What is the blood Phe level cut-off for a positive neonatal screening test in your country?							N/A
120 μmol/L	180 μmol/L	240 μmol/L	360 μmol/L	Other^a^		No answer	
48.4 %	25.8 %	9.7 %	3.2 %	9.7 %		3.2 %	
For which patient age groups does your centre perform the BH4 loading test?^b^							N/A
Newborns	Infants	Young children	Older children	Adolescents	Adults	No answer	
38.7 %	9.7 %	25.8 %	32.3 %	22.6 %	3.2 %	35.5 %	
What is the duration of the BH4 loading test?							N/A
8 h	24 h	48 h	72 h	7 days	30 days	No answer	
0 %	35.5 %	9.7 %	9.7 %	0 %	3.2 %	41.9 %	
What percentage of patients at your centre are BH4 responders?							15 (0–31)
0 %	1–5 %	6–10 %	11–20 %	≥21 %	No answer		
3.2 %	9.7 %	6.5 %	9.7 %	12.9 %	58.1 %		

*BH4* sapropterin dihydrochoride, *N/A* not applicable, *Phe* phenylalanine

^a^‘Other’ was specified by three questionnaires as 80, 132 and 150 μmol/L

^b^Multiple answers were possible, and therefore, the total exceeds 100 %

After a positive newborn screening test, diagnosis was confirmed by tandem mass spectrometry (39 %), amino acids chromatography (26 %), and/or a fluorescent, enzymatic or colorimetric method (58 %). No centres reported relying on the Guthrie test for confirmatory diagnosis. Routine genetic analysis was performed at 64.5 % of centres as part of their diagnostic procedure (centres in Croatia (1/1), Romania (3/3), Turkey (5/7) and Poland (1/7) did not conduct genetic analysis). Of the centres, 51.6 % performed routine sapropterin dihydrochloride (tetrahydrobiopterin (BH4)) loading tests (newborns, 38.7 %; older children, 32.3 %; adults, 3.2 %). This test is used to investigate a diagnosis of BH4-responsive PKU and/or to rule out BH4 deficiency in newborns. At these centres, the BH4 loading test dose was 20 mg/kg, BH4 responsiveness in PKU was consistently defined as a reduction in Phe concentration of ≥30 %, and the most common duration of the test was 24 h (35.5 %; range 24 h–30 days). A median of 15 % (range 0–31) of patients were classified as BH4 responders. Other tests used to distinguish BH4 deficiency from PKU included pterins and dihydropteridine reductase (DHPR) analysis (45.2 %).

### Treatment practices and reimbursement

All centres advocated life-long treatment with a low-Phe diet (based on Phe-free amino acid supplements and special low-protein foods) (Table 3), whereas BH4 and large neutral amino acids were available in 48 and 32 % of centres, respectively (in Poland BH4 treatment was only available for patients with BH4 deficiency).

**Fig. 2:**
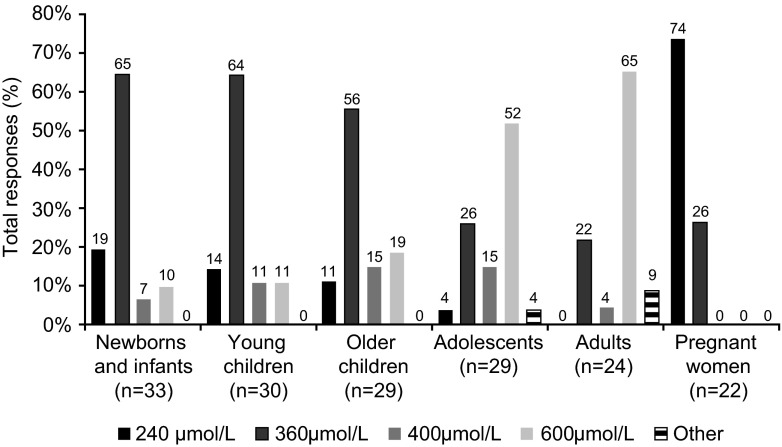
Blood Phe threshold levels for starting low-Phe diet treatment in each patient group (consistently elevated levels), ‘*n*’ represents the number of questionnaires which provided an answer for each group (total responses); ‘Other’ was specified for adolescents as ‘480 μmol/L’, and for adults as ‘130–900’ and ‘900 μmol/L’; age groups were as follows: newborns and infants, <1 year of age; young children, 1–4 years of age; older children, 5–10 years of age; adolescents, 11–17 years of age; adults, ≥18 years of age

Blood Phe thresholds for starting low-Phe diet treatment in different patient groups are presented in Fig. 2. For newborns, the most common blood Phe threshold for initiation of treatment was ≥360 μmol/L (65 %; other thresholds used were ≥240 μmol/L [19 %], ≥400 μmol/L [6 %], ≥600 μmol/L [10 %]). For young and older children, this was also ≥360 μmol/L (64 and 56 %, respectively), whereas for adolescents and adults, it was ≥600 μmol/L (52 and 65 %, respectively). Phe thresholds to commence treatment were not always the same between centres in the same country (data not shown).

**Fig. 3:**
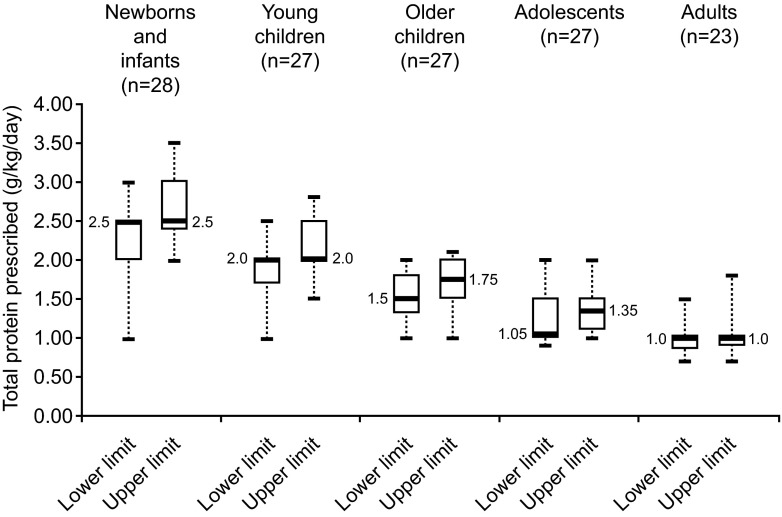
Dose of total protein (g/kg/day) prescribed to patients with classical PKU by each centre per patient age group. Box and whisker plot, with *boxes* representing interquartile range (*thick line* indicating the median, with value indicated in the plot), *dashed whiskers* minimum and maximum values. ‘*n*’ represents the number of questionnaires which provided an answer for each group; age groups were as follows: newborns and infants, <1 year of age; young children, 1–4 years of age; older children, 5–10 years of age; adolescents, 11–17 years of age; adults, ≥18 years of age

Seventy-one percent of centres started administering low-Phe diet treatment before 15 days of a patient’s life, with 26 % reporting this to be >15 days (Table 3). The median dose of total protein (natural and Phe-free L-amino acid supplement) prescribed to newborns and infants with classical PKU was 2.5 g/kg/day (median [range] lower limit 2.50 [1.0–3.0] g/kg/day, median upper limit 2.50 [2.0–3.5] g/kg/day), which decreased steadily with increasing patient age to 1 g/kg/day in adults (median [range] lower limit 1.0 [0.7–1.5] g/kg/day, median upper limit 1.0 [0.7–1.8] g/kg/day) (Fig. 3).

Low-Phe diet was available to treat maternal PKU at all centres, although only 22 centres indicated in their questionnaire the Phe level thresholds used to initiate treatment in maternal PKU. This was either ≥240 μmol/L (74 %) or ≥360 μmol/L (26 %; Fig. 2).

With respect to reimbursement and social support, governments contributed to costs of Phe-free L-amino acid supplements in the majority of centres (97 %), although there was less governmental support to cover costs of special low-protein foods (59 %; Table 3). Patients at most centres were entitled to a disability allowance and/or a disability certificate to cover out of pocket costs (81 %), although there was no social support available for patients at three centres (one in each of Bulgaria, Hungary and Turkey) (Table 3).

**Table 3:** Summary of treatment and reimbursement information (*N* = 31)

What treatment options are available in your country?					
Low-Phe diet	BH4	Large neutral amino acids		Other^a^	
100 %	48.4 %	32.3 %		3.2 %	
At what age does your centre introduce low-Phe diet in newly diagnosed newborns?					
0–9 days	10–15 days	16–28 days	≥29 days	No answer	
19.4 %	51.6 %	22.6 %	3.2 %	3.2 %	
Who contributes to the costs of Phe-free protein substitutes?^b^					
Government		Private health insurance		Parents/patients	
96.8 %		12.9 %		22.6 %	
Who contributes to the cost of special low-protein foods (flour, pasta)?^b^					
Government	Private health insurance	Parents/patients		Other^c^	
59.4 %	12.5 %	75.0 %		6.3 %	
What kind of social support is offered to PKU patients in your country?^b^					
Disability allowance	Disability certificate which helps with education, employment, travel expenses	Dietary allowance to go to summer camps	Reimbursement of travel expenses	Home support or compensation to parents for decreasing working hours	No support available
80.6 %	45.2 %	32.3 %	25.8 %	12.9 %	9.7 %

*BH4* sapropterin dihydrochoride, *Phe* phenylalanine, *PKU* phenylketonuria

^a^‘Other’ was specified as ‘BH4 only for BH4-deficient patients’

^b^Multiple answers were possible, and therefore, the total exceeds 100 %

^c^‘Other’ was specified as: ‘sellers’, ‘non-governmental organisation’ and ‘parents/patients association’

### Follow-up practices

A specific follow-up protocol for patients with PKU was used in 77.4 % of centres (Table 4). Most centres collected blood samples from the patient’s home (74.2 %) and in outpatient clinics (67.7 %), and 61 % returned blood Phe results within 4 days (range 1–8 days), using e-mail, phone, letter and clinic visits (data not shown).

**Table 4:** Summary of follow-up practices (*N* = 31)

Question and answers by category						Median (range)
Does your centre follow a specific follow-up protocol for PKU patients?						N/A
Yes		No		No answer		
77.4 %		19.4 %		3.2 %		
Where does your centre collect samples for monitoring Phe levels in treated PKU patients?^a^						N/A
Home	Outpatient clinic	Hospital		Family doctor clinic (general practitioner)		
74.2 %	67.7 %	35.5 %		29.0 %		
What is the average return time of routine Phe control results to your patients, after blood sampling?						N/A
1–2 days	3–4 days	5–6 days	7–8 days	No answer		
25.8 %	35.5 %	16.1 %	19.4 %	3.2 %		
What proportion of your patients is lost to follow-up (defined as patient was not seen for two years)?						10 (0–21)
0 %	1–5 %	6–10 %	11–20 %	≥21 %	No answer	
9.7 %	22.6 %	22.6 %	25.8 %	3.2 %	16.1 %	

*N/A* not applicable, *Phe* phenylalanine, *PKU* phenylketonuria

^a^Multiple answers were possible, and therefore, the total exceeds 100 %

The median percentage of patients ‘lost to follow-up’ (as defined by a patient not being seen for over 2 years) was 10 %. There was a general trend for higher lost to follow-up rates in the larger centres. Among the centres providing this data, the 13 largest centres (range of patients 130–2500) had a median of 15 % patients lost to follow-up, whereas this was 5 % for the 13 smallest centres (range of patients 3–123).

**Fig. 4:**
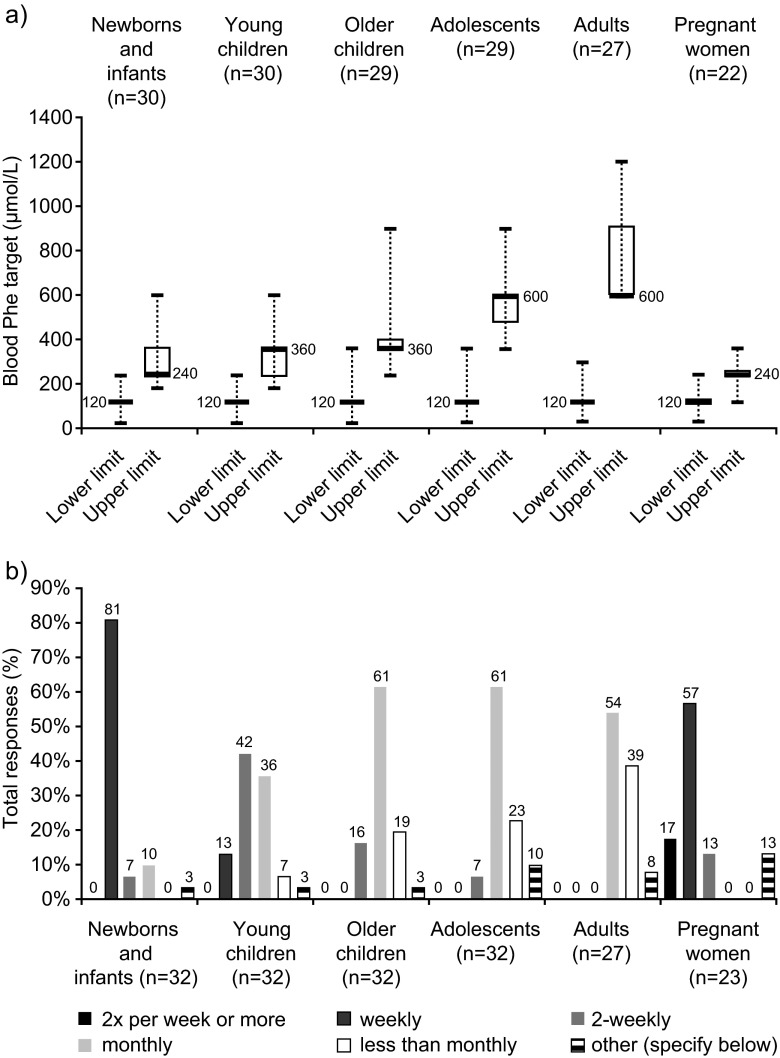
Blood Phe target ranges (μmol/L) and frequency of blood Phe monitoring for different patient groups. **a** Box and whisker plot of blood Phe target ranges per patient group: *boxes* represent interquartile range (*thick line* indicating the median, with value indicated in the plot), and *dashed whiskers* minimum and maximum values. **b** Frequency histogram of blood Phe monitoring frequency. ‘Other’ was as follows: newborns and infants: ‘sometimes weekly, sometimes 2-weekly’; young children: ‘1–2 years: 2-weekly; 2–3 years: 3-weekly; 3–4 years: monthly’; older children: not specified; adolescents: ‘2-weekly or monthly’ ‘depends on patient collaboration’; not specified; adults: ‘It depends on the patient collaboration’, not specified; maternal PKU: ‘sometimes weekly, sometimes 2X per week’; not specified (2). ‘n’ represents the number of questionnaires which provided an answer for each group. Age groups were as follows: newborns and infants, <1 year of age; young children, 1–4 years of age; older children, 5–10 years of age; adolescents, 11–17 years of age; adults, ≥18 years of age

Blood Phe level target ranges are presented in Fig. 4a. With a few exceptions, the lower Phe target was 120 μmol/L across all age groups. The median (range) upper Phe level target was 240 μmol/L (180–600) in newborns, 360 μmol/L (180–900) in children, and 600 μmol/L (360–1200) in teenagers and adults. Upper Phe targets were more variable across centres than lower Phe levels targets, with the interquartile range spanning >100 μmol/L for newborns and infants, young children and adults. With respect to the frequency of Phe monitoring, there was a general trend towards less frequent monitoring with increasing patient age (Fig. 4b).

In maternal PKU, the median (range) of the upper blood Phe target while on treatment was 240 μmol/L (120–360) (Fig. 4a), and Phe level monitoring was most commonly on a weekly basis (13/23; 57 %), with 3/23 (13 %) centres monitoring every 2 weeks (Fig. 4b).

### Guidelines, registries and organisations devoted to PKU

A variety of guidelines and protocols for PKU diagnosis and treatment were in use across the centres: while 32 % of centres used only published guidelines/protocols, 32 % used only their own unpublished guidelines/protocols and 29 % of centres used a mixture of both types. Eighty-five percent of centres were aware of either a local or national PKU registry, and 94 % were aware of patient/family organisations.

### Challenges and areas for improvement

Participants were asked to describe the main challenges that they face in terms of screening, diagnosis and treatment of PKU; a summary of this feedback is presented in Online Resource 3.

### Centres providing demographic data only/e-mail responses

Four centres (Bosnia and Herzegovina, Republic of Macedonia, Romania and Serbia) provided only demographic data. These centres collectively cared for 27 patients with PKU, a high proportion of whom were late diagnosed (range 20–90 % of patients). The Republic of Macedonia was previously reported to have no newborn screening for PKU [9], and the contacted centre indicated via e-mail that they manage the sporadic cases they encounter with an occupational therapy plan.

Two centres (Albania and Montenegro) provided only short responses by e-mail, each reporting that there was no PKU screening in their respective countries.

## Discussion

### Challenges in diagnosis and treatment

A well-documented issue for PKU care is that management guidelines differ between countries, often in important areas such as newborn screening, target blood Phe levels in different patient groups, frequency of Phe monitoring and duration of treatment [29]. Our survey adds to a growing body of evidence showing a difference in PKU care between Western and South and Eastern Europe and provides further reason to support efforts to raise and standardise treatment across Europe.

The most critical disparity concerns the lack of newborn screening in several centres, thereby leading to late commencement of treatment. Results from this survey and complementary studies [9, 11] highlight that although newborn screening is widespread, it is not yet implemented in all European countries. This includes five of the target countries of this survey, namely Albania, Kosovo (from where no information was returned), Malta, Montenegro and the Republic of Macedonia (Fig. 1). Furthermore, in some countries with established newborn screening programmes, including Bulgaria and Romania, up to 10 % of newborns are not screened [9]. Based on these data and current birth rates [6, 9], there were approximately 170,000 live births unscreened in 2013 across the target countries of this survey. Considering an estimated PKU prevalence of 1/10,000 live births [16], 17 newborns per year may face a delayed diagnosis across these countries.

Our results from 31 centres providing complete questionnaires (of which 30 had newborn screening) suggest that diagnostic testing is not always comprehensive or optimally managed in the region. For example, fewer than half of the centres reported using specific tests, such as pterins and DHPR analysis, to diagnose BH4 deficiency. Also, the routine use of BH4 loading tests was linked to the availability of BH4 treatment for patients with PKU, and a lack of BH4 testing and treatment were highlighted as main challenges/areas for improvement (Online Resource 3). Nevertheless, use of some advanced diagnostic techniques was surprisingly widespread: genetic analysis was routinely used in 13 of the countries represented in this survey, higher than reported elsewhere [1].

Another concern is the time taken for positively screened patients to be seen at some centres, which was over 2 weeks in 20 % of centres and over 1 month in one centre, and was mirrored by a similar delay in starting treatment at these centres. By contrast, US guidelines recommend that initiation of treatment for PKU should preferably occur within the first week of life, with the aim to have achieved target blood Phe concentrations within the first 2 weeks of life [30].

Regarding the professionals within the treatment teams, there was a notable lack of dedicated dieticians in 30 % of the centres in this survey (compared with only 6 % in the predominantly Western European survey [1]). Dieticians are key to ensuring best management of the complex dietary requirements of patients with PKU [7, 17]. The general lack of all core professionals within treatment teams and of specialised adult PKU clinics has also been reported elsewhere, including in countries with advanced management approaches [1, 26, 27].

A striking statistic was the relatively large number of patients per centre who lived within large catchment areas. There were approximately two times more patients per centre than in a predominantly Western European survey [1], and the median travel distance to the centre was over 100 km. This could impact care in two ways. Firstly, an increased patient load may put increased strain on resources (additional information on patient staffing levels will be needed to assess this possibility, see ‘Sect. Limitations of study’). Secondly, living further than approximately 160 km from the care centre has been associated with significantly fewer Phe samples for analysis [8], which raises the possibility that distance may impact outcomes. The proportion of patients lost to follow-up (median of 10 % in this survey), might also be influenced by the size of these catchment areas and patient loads. This situation might be caused by countries adopting a ‘tertiary referral hospital’ model, which might not be optimal in all countries. Notably, it could add to out-of-pocket costs of patients travelling to the centres. Individual countries should review whether it is more effective to redistribute resources at a more local level for managing PKU care.

Commencement and management of treatment was also not always optimal. Inconsistencies across centres in the Phe level thresholds to start treatment and upper Phe level targets during treatment is a universal issue [1, 11] and may lead to different outcomes for patients with an otherwise similar condition [12]. Monitoring frequency followed the expected trend of being most frequent in newborns and declining with increasing patient age, although this was also variable across centres. Importantly, in maternal PKU, 10 % of centres monitored Phe levels only once every 2 weeks, which is sub-optimal given the changing Phe requirements during pregnancy and risks associated with both high and low Phe concentrations: very low levels are associated with intrauterine growth retardation; high levels (>360 μmol/L) carry a high risk of maternal PKU syndrome [13, 25]. It is hoped that several of these issues will be addressed in the near future by the release of Pan-European guidelines [11].

A lack of treatment options (i.e. availability of BH4, variety of protein substitutes and foods) featured as a main challenge, as noted elsewhere [1, 11]. About half of centres relied only on low-Phe diet, and some felt that the variety of Phe-free protein substitutes and low-protein foods available to patients was limited due to a lack of reimbursement. These issues could decrease adherence to a low-Phe diet [18]. Given the relatively low income of many families in the surveyed countries, the cost and availability of low-Phe foods is an important issue that must be addressed by healthcare systems and governments. BH4 treatment is expected to become more widely available in the coming years, although reimbursement issues are likely to affect its impact.

### Limitations of study

This analysis is subject to several limitations. Unfortunately, of the five countries known to lack newborn screening, only one centre in Malta provided a completed questionnaire. Centres in The Republic of Macedonia, Albania and Montenegro provided limited information and no response was received from Kosovo, meaning that we were unable to comprehensively evaluate standard of care in these locations. The number of patients with PKU in the region who remain undiagnosed is also unknown and is likely to be a major issue in areas where newborn screening was only introduced recently (e.g. in Romania, nationwide newborn screening was only introduced in 2011 [9]). Thus, there may be severely intellectually disabled patients living without a correct diagnosis or appropriate medical care not recognised by this survey [19, 21, 28].

Several targeted centres did not answer the questionnaire. Literature-based estimates put the population with PKU in the surveyed region at around 19,000 patients [16], which suggests a 45 % coverage by this survey. A lack of translation into the local language or poor access to computers and/or the internet may have hampered questionnaire completion by some centres. Additionally, contacts were lacking for Belarus, and the list of contacts for other countries may not have been exhaustive (although in an additional question, the 31 centres in the main results section indicated that a cumulative total of 45 centres existed across their countries, comparable to the 46 that were contacted, so it is likely that very few were omitted; see Online Resource 1 and 2).

The centres in this survey care for different numbers of patients with PKU, and therefore, some apply a management approach which affects far more patients than others (whereas the size of each centre is not weighted in the above analysis). It should also be noted that neither the extent to which each member of the treatment team is dedicated to PKU, nor staff to patient ratios were established. These two factors play an important role in the delivery of care.

Finally, the questionnaire used non-validated questions, which were open to misinterpretation.

## Conclusion

Results of this survey point to several important areas for improvement in PKU diagnostic and management practices across Southern and Eastern Europe. Given that there remain parts of Europe where there is no routine newborn screening (or it has only been recently introduced), older patients with PKU who can benefit from low-Phe diet treatment [14, 31] are likely under-diagnosed. Interestingly, coverage of newborn screening and diagnostic techniques may not necessarily be related to the economic standing of the country [9]. Furthermore, the diagnostic and treatment packages offered to patients may be more influenced by the interests and skills of the PKU teams rather than the direct needs of the patients. New evidence-based Pan-European guidelines are currently in development with the aim of encouraging a common standard of care [11]. It is important that development of new guidelines coincides with efforts by HCPs and governments across the region to ensure all patients receive the best possible care.

### Electronic supplementary material

ESM. 1 (PDF 559 kb)

ESM. 2 (PDF 644 kb)

ESM. 3 (PDF 445 kb)

## Appendix

Contributing HCPs and centres.

Bulgaria: Radka Tincheva, Aleksei Savov, Adil Kadam (University Pediatric Hospital Sofia); Croatia: Vladimir Sarnavka, Ivo Barić (University Hospital Centre Zagreb); Czech Republic: Dagmar Prochazkova (I.dětská interní klinika FN Brno), Renata Pazdírková, Jana Komárková, Hana Kothánková (Klinika dětí a dorostu FNKV, Praha); Estonia: Katrin Ounap, Karit Reinson, Mari-Liis Uudelepp (Tartu University Clinicum, Tartu); Hungary: László Szőnyi, Erika Kiss, Péter Reismann (Semmelweis University, Hungary); Latvia: Rita Lugovska, Parsla Vevere (Children clinical university hospital, Riga); Lithuania: Loreta Cimbalistiene (Vilnius University, Vilnius); Malta: Simon Attard Montalto (Mater Dei Hospital, University of Malta); Poland: Agnieszka Chrobot, Izabela Horka (Bydgoszcz), Bożena Didycz, Mirosław Bik-Multanowski (Kraków), Bożena Mikołuć, Maria Jolanta Piotrowska-Depta, Ewa Samocik (Białystok), Ewa Starostecka, Agata Lange (Łódź), Jolanta Wierzba, Joanna Jagłowska (Gdańsk), Kalina Plutowska-Hoffmann, Joanna Zarębska (Katowice), Maria Giżewska, Hanna Romanowska, Elżbieta Krzywińska-Zdeb (Szczecin), Maria Nowacka, Joanna Żółkowska, Dorota Korycińska-Chaaban (Warszawa); Republic of Moldova: Usurelu Natalia (Institute of Mother and Child, Chisinau); Romania: Anton-Paduraru Dana-Teodora (Spitalul clinic de urgenta pentru copii “Sf. Maria”, Iași), Mariana Muresan (Clinica de Pediatrie III, Cluj Napoca), Nanu Michaela Iuliana, Moldovanu Florentina, Iorgulescu Daniela (IOMC Bucuresti, Bucharest); Serbia: Maja Đorđević, Božica Kecman, Adrijan Sarajlija (Institut za zdravstvenu zaštitu majke i deteta Srbije, Novi Beograd), Slovakia: Katarína Hálová (Detská fakultná nemocnica, Banska Bystrica); Slovenia: Mojca Zerjav Tansek (University Clinical Center Ljubljana); Turkey: Burcu Öztürk Hişmi (Çocuk Metabolizma Hastalıkları ve Beslenme Uzmanı Gaziantep Çocuk, Gaziantep); Serap Sivri (Hacettepe University Faculty of Medicine, Hacettepe); Isil Ozer (Medeniyet University, Istanbul, Turkey); Mahmut Coker, Sema Kalkan Ucar (Ege University, Izmir); Neslihan önenli Mungan, Deniz Kör, Berna şeker Yilmaz (Cukurova University, Adana), Nur Arslan, Yesim Ozturk (Dokuz Eylul University, Izmir); Selda Bulbul (Kirikkale University, Turkey).

Centres providing e-mail/incomplete questionnaire responses.

Albania: Lindita Grimci (University Hospital Center “Mother Teresa”, Tirana); Bosnia and Herzegovina: Smail Zubcevic (Clinical Center University of Sarajevo, Faculty of Medicine); Montenegro: Mira Samardzic (Institute for Sick Children, Podgorica); Republic of Macedonia: Elena Sukarova Angelovska, Natalija Angelkova (University Children’s Hospital Skopje); Romania: Otilia Marginean, Marinela Lesovici (Spitalului Clinic de Urgenta Pentru Copii “Louis Turcanu”, Timisoara); Serbia: Jovanovic Privrodski Jadranka, Kavecan Ivana (Institut Za Decu i Omladinu, Novi Sad).

## Abbreviations

BH4: Tetrahydrobiopterin or sapropterin dihydrochloride
DHPR: Dihydropteridine reductase
HCP: Health care professional
N/A: Not applicable
Phe: Phenylalanine
PKU: Phenylketonuria
