# Supplementary material for: Diagnostic and management practices for phenylketonuria in 19 countries of the South and Eastern European Region: survey results
Source: Eur J Pediatr. 2015 Sep 8;175:261–72. doi: 10.1007/s00431-015-2622-5 (PMC4724370; doi:10.1007/s00431-015-2622-5)
Supplement: Supplementary file 1 — (PDF 559 kb) [file 431_2015_2622_MOESM1_ESM.pdf]

Article title: Diagnostic and management practices for phenylketonuria in 19 countries of the South and Eastern European Region: survey results

Journal: European Journal of Pediatrics

Authors: Maria Giżewska, Anita MacDonald, Amaya Bélanger-Quintana, Alberto Burlina, Maureen Cleary, Turgay Coşkun, François Feillet, Ania C. Muntau, Friedrich Trefz, Francjan van Spronsen, Nenad Blau

Corresponding author: Maria Giżewska, Pomeranian Medical University, Szczecin, Poland, maria.gizewska@gmail.com

Online resource 1

Full questionnaire emailed to health care professionals. Questions marked with * were mandatory.

1. General information

***a. Please provide the names of all people from your centre taking part in this survey (as you would like the names to appear when published in the acknowledgments):**

Participant 1. [ ]

Participant 2. [ ]

Participant 3. [ ]

***b. Participant 1. What is your function? (please select your primary role)**

- ◯ Physician
- ◯ Paediatrician
- ◯ Clinical geneticist
- ◯ Metabolic paediatrician
- ◯ Adult metabolic doctor
- ◯ Gynaecologist/obstetrician (maternal PKU)
- ◯ Dietician/Nutritionist
- ◯ Dietician, metabolic diseases
- ◯ Nurse specialising in PKU
- ◯ Psychologist
- ◯ Clinical biochemist
- ◯ Research scientist
- ◯ Other (please specify) [ ]

Participant 2. What is your function? (please select your primary role)

- ○ Physician
- ○ Paediatrician
- ○ Clinical geneticist
- ○ Metabolic paediatrician
- ○ Adult metabolic doctor
- ○ Gynaecologist/obstetrician (maternal PKU)
- ○ Dietician/Nutritionist
- ○ Dietician, metabolic diseases
- ○ Nurse specialising in PKU
- ○ Psychologist
- ○ Clinical biochemist
- ○ Research scientist
- ○ Other (please specify)

Participant 3. What is your function? (please select your primary role)

- ○ Physician
- ○ Paediatrician
- ○ Clinical geneticist
- ○ Metabolic paediatrician
- ○ Adult metabolic doctor
- ○ Gynaecologist/obstetrician (maternal PKU)
- ○ Dietician/Nutritionist
- ○ Dietician, metabolic diseases
- ○ Nurse specialising in PKU
- ○ Psychologist
- ○ Clinical biochemist
- ○ Research scientist
- ○ Other (please specify)

***c. How many years of experience do you have with PKU diagnosis and treatment?**

Partcipant 1. Number of years:

Partcipant 2. Number of years:

Partcipant 3. Number of years:

***d. Participant 1. With which group(s) of PKU patients are you involved? (multiple answers possible)**

- ☐ All patients with PKU
- ☐ Newborns, children and teenagers (<18 years of age)
- ☐ Adults (≥18 years of age)
- ☐ Maternal PKU
- ☐ PKU patients with intellectual disability
- ☐ Other (please specify)

Participant 2. With which group(s) of PKU patients are you involved? (multiple answers possible)

- ☐ All patients with PKU
- ☐ Newborns, children and teenagers (<18 years of age)
- ☐ Adults (≥18 years of age)
- ☐ Maternal PKU
- ☐ PKU patients with intellectual disability
- ☐ Other (please specify)

Participant 3. With which group(s) of PKU patients are you involved? (multiple answers possible)

- ☐ All patients with PKU
- ☐ Newborns, children and teenagers (<18 years of age)
- ☐ Adults (≥18 years of age)
- ☐ Maternal PKU
- ☐ PKU patients with intellectual disability
- ☐ Other (please specify)

e. How many centres or clinics offer a PKU service in your country? (leave blank if you do not know)

Number of centres and clinics:

Please list centres (and, if known, name of contact person) here:

Centre and Contact:

Centre and Contact:

Centre and Contact:

Centre and Contact:

Centre and Contact:

Centre and Contact:

Centre and Contact:

Centre and Contact:

Centre and Contact:

Centre and Contact:

f. Approximately how many PKU patients (treated and untreated) are there in your country? (leave blank if you do not know)

Number of patients:

g. How many PKU patients are currently followed at your clinic/centre? (If you do not know exactly, please provide your best estimate)

Number of patients:

h. Please approximate the number of PKU patients followed over the course of a typical (i.e. average) year at your clinic/centre, as follows: (leave blank if you do not know)

Number of new patients per year:

Total number of patients per year (including the new patients):

i. Please approximate the distribution of all PKU patients followed over the course of a typical (i.e. average) year at your clinic/centre, as follows: (leave blank if you do not know)

Percentage of children (<18 years of age):

Percentage of adults (≥18 years of age):

j. Approximately how many maternal PKU pregnancies are followed over the course of a typical (i.e. average) year at your clinic/centre? (leave blank if you do not know)

Number of patients:

k. <u>Approximately</u> what percentage of PKU patients followed at your centre are lost to follow-up (defined as: patient was not seen for 2 years)? (leave blank if you do not know)

Percentage of patients:

l. <u>Approximately</u> what percentage of PKU patients currently followed at your centre were late diagnosed (meaning not as newborns)? (leave blank if you do not know)

Percentage of patients:

m. <u>Approximately</u> what is the average distance (km) that PKU patients currently have to travel to your centre? (leave blank if you do not know)

Number of kilometers:

n. <u>Approximately</u> how many PKU patients at your centre are followed in another country? (leave blank if you do not know)

Number of patients:

2. Screening and confirmatory diagnosis procedures:

a. Is neonatal screening available in your country?

- ◯ Yes
- ◯ No

If yes, is the programme public or private?

- ◯ Public
- ◯ Private
- ◯ Partially public, partially private

If yes, does it exist at a regional or national level?

- ◯ At a regional level:
- ◯ At a national level:

b. When was neonatal screening implemented? (please provide year as YYYY; eg. 2013)

At a regional level [ ]

At a national level [ ]

c. Is neonatal screening mandatory for all newborns in your centre?

- ◯ Yes
- ◯ No

If no, what % of all newborns are screened at your centre?

[ ]

d. At what age is the heel prick test performed at your centre?

Age (days): [ ]

e. At what age are positively screened newborns seen in the medical service?

Age (days): [ ]

f. What is the blood Phe level cut-off for a positive neonatal screening test in your country?

Cut-off (μmol/L): [ ]

or Cut-off (mg/dL): [ ]

g. What is the method used to confirm a positive neonatal PKU screening test at your centre? (multiple answers possible)

- [ ] Guthrie method
- [ ] Colorimetric method
- [ ] MS/MS
- [ ] HPLC
- [ ] Other (please specify)

h. Who contacts parents/patients following a positive neonatal PKU screening test at your centre? (multiple answers possible)

- [ ] Physician
- [ ] Paediatrician
- [ ] Clinical geneticist
- [ ] Metabolic paediatrician
- [ ] Dietician/Nutritionist
- [ ] Dietician, metabolic diseases
- [ ] Nurse specialising in PKU
- [ ] Psychologist
- [ ] Clinical biochemist
- [ ] Research scientist
- [ ] Other (please specify)

i. What is the method used to contact parents/patients following a positive neonatal PKU screening test at your centre? (multiple answers possible)

- [ ] Telephone
- [ ] Email
- [ ] Other (please specify)

j. Is the confirmatory diagnosis of PKU and introduction of treatment performed for in-patients or out-patients at your centre? (multiple answers possible)

- [ ] In-patients (i.e. patients admitted to the hospital and staying > 24 hours)
- [ ] Out-patients (i.e. patients attending a clinic and staying at the hospital < 24 hours)

k. Does your centre routinely perform sapropterin dihydrochloride (BH4) loading tests?

- ◯ Yes
- ◯ No

l. For which patient age groups does your centre perform the BH4 loading test? (multiple answers possible; leave blank if your centre does not perform BH4 loading tests)

- ☐ Newborns (0–28 days)
- ☐ Infants (29 days–<1 year of age)
- ☐ Young children (1–4 years of age)
- ☐ Older children (5–10 years of age)
- ☐ Teenagers (11–17 years of age)
- ☐ Adults (≥18 years of age)
- ☐ Other (please specify)

m. If a BH4 test is routinely used, please indicate the dosage:

Dosage mg/kg/day

n. If a BH4 test is routinely used, please indicate the duration of the test:

- ☐ 8 hours
- ☐ 24 hours
- ☐ 48 hours
- ☐ 72 hours
- ☐ 7 days
- ☐ 30 days
- ☐ Other (please specify)

o. If your centre performs BH4 loading tests, are they generally done before starting with a low-phenylalanine (Phe) diet?

- ◯ Yes
- ◯ No

p. What reduction in Phe concentration (%) does your centre use to define BH4 responsiveness?

Phe concentration reduction (%):

q. What percentage of patients at your centre are BH4 responders? (leave blank if you do not know)

Patients (%): __________

r. Does your centre routinely perform pterins analysis?

- ◯ Yes
- ◯ No

If yes, what is the method used for pterins analysis? (multiple answers possible)

- ☐ in urine
- ☐ in dry blood spot

s. Does your centre routinely perform DHPR activity analysis?

- ◯ Yes
- ◯ No

t. Does your centre routinely perform genotype analysis?

- ◯ Yes
- ◯ No

If yes, would the information be included in the BIOPKU database (www.biopku.org)?

- ◯ Yes
- ◯ No
- ◯ I am not aware of this database

3. Treatment practices

a. What treatment options are available in your country? (multiple answers possible)

- [ ] Low-Phe diet
- [ ] BH4
- [ ] Large neutral amino acid
- [ ] Other (please specify)

b. What are the blood Phe threshold levels for starting low-Phe diet treatment in each patient group at your centre?

	Consistently elevated blood Phe levels of:
Newborns and infants (<1 year of age)	
Young children (1–4 years of age)	
Older children (5–10 years of age)	
Teenagers (11–17 years of age)	
Adults (≥18 years of age)	
Pregnant women	

Specify here if other blood Phe threshold levels are used for each patient group

c. At what age does your centre introduce low-Phe diet in newly diagnosed newborns?

- <10 days
- 10-15 days
- 16-28 days
- ≥29 days

d. Does your centre allow breastfeeding in neonates and infants with PKU?

- Yes
- No

e. Life-long concept of PKU treatment is:

- ◯ Used at all PKU centres in your country
- ◯ Used at your centre, but not neccesarily at all PKU centres in your country
- ◯ Not used at your centre, but might be available at other PKU centres in your country
- ◯ Not used anywhere in your country

f. Treatment with low-Phe diet to prevent maternal PKU syndrome is:

- ◯ Available at all PKU centres in your country
- ◯ Available at your centre, but not neccesarily at all PKU centres in your country
- ◯ Not available at your centre, but might be available at other PKU centres in your country
- ◯ Not available anywhere in your country

g. Treatment with low-Phe diet for untreated or late-treated PKU patients is:

- ◯ Available at all PKU centres in your country
- ◯ Available at your centre, but not neccesarily at all PKU centres in your country
- ◯ Not available at your centre, but might be available at other PKU centres in your country
- ◯ Not available anywhere in your country

h. BH4 treatment is:

- ◯ Available at all PKU centres in your country
- ◯ Available at your centre, but not neccesarily at all PKU centres in your country
- ◯ Not available at your centre, but might be available at other PKU centres in your country
- ◯ Not available anywhere in your country

i. For patients with classical PKU, what dose of <u>total</u> protein (g/kg/day) does your centre prescribe for the following age groups? (Please specify the min-max range using g/kg/day, using a decimal point if required)

Newborns and infants (<1 year of age) (g/kg/day)	
Young children (1–4 years of age) (g/kg/day)	
Older children (5–10 years of age) (g/kg/day)	
Teenagers (11–17 years of age) (g/kg/day)	
Adults (≥18 years of age) (g/kg/day)	

j. Do PKU patients at your centre take any of the additional supplements (besides in Phe-free protein substitutes) on the following list? (Multiple answers are possible; if the answer is no, leave blank)

- [ ] Long chain polyunsaturated fatty acids
- [ ] Vitamins and minerals
- [ ] Carnitine
- [ ] Antioxidants
- [ ] Cod liver oil
- [ ] Other supplements (please specify)

4. Follow-up

a. Does your centre follow a specific "follow-up protocol" for PKU patients?

- ◯ Yes
- ◯ No

b. What are the blood Phe target ranges for each PKU patient group at your centre? (Please specify the min–max range using either µmol/L or mg/dL, and use a decimal point if required)

Newborns and infants (<1 year of age), range µmol/L:

or range mg/dL:

Young children (1–4 years of age), range µmol/L:

or range mg/dL:

Older children (5–10 years of age), range µmol/L:

or range mg/dL:

Teenagers (11–17 years of age), range µmol/L:

or range mg/dL:

Adults (≥18 years of age), range µmol/L:

or range mg/dL:

Maternal PKU, range µmol/L:

or range mg/dL:

c. How often are Phe levels monitored at your centre in each PKU patient group?

	Frequency of monitoring
Newborns and infants (<1 year of age)	
Young children (1–4 years of age)	
Older children (5–10 years of age)	
Teenagers (11–17 years of age)	
Adults (≥18 years of age)	
Maternal PKU	

specify here if other frequencies are used for each patient group:

d. Where does your centre collect samples for monitoring Phe levels in treated PKU patients? (multiple answers possible)

- [ ] Home
- [ ] Outpatient clinic
- [ ] Hospital
- [ ] Family doctor clinic (general practitioner)

e. Approximately what is the average return time of routine Phe control results to your patients, after blood sampling?

Number of days: ________

f. How do patients at your centre receive results of Phe levels? (multiple answers possible)

- [ ] E-mail
- [ ] Phone
- [ ] Letter
- [ ] Clinic visits
- [ ] Other (please specify)

g. When giving the Phe results, who contacts parents/patients to provide advice or make changes to diet or other treatment? (multiple answers possible)

- [ ] Physician
- [ ] Paediatrician
- [ ] Clinical geneticist
- [ ] Metabolic paediatrician
- [ ] Adult metabolic doctor
- [ ] Dietician/Nutritionist
- [ ] Dietician, metabolic diseases
- [ ] Nurse specialising in PKU
- [ ] Psychologist
- [ ] Clinical biochemist
- [ ] Research scientist
- [ ] Other (please specify)

h. How many clinical evaluations are performed per year in children and non-pregnant adults with PKU?

Newborns and infants (<1 year of age)

Young children (1–4 years of age)

Older children (5–10 years of age)

Teenagers (11–17 years of age)

Adults (≥18 years of age)

Maternal PKU

i. In pregnant women with PKU (maternal PKU), what is the frequency of clinical evaluations per trimester of pregnancy?

	Once every week	Once every two weeks	Once every month	Other (specify below)
First trimester	○	○	○	○
Second trimester	○	○	○	○
Third trimester	○	○	○	○

Specify here if other frequencies are used in each trimester

j. Who cares for PKU patients from the age of 18 years? (multiple answers possible)

- [ ] PKU paediatric clinic
- [ ] PKU adult clinic
- [ ] Other (please specify)

5. Constitution of treatment team

a. The PKU team at your centre includes which of the following? (multiple answers possible)

- [ ] Physicians
- [ ] Paediatricians
- [ ] Clinical geneticists
- [ ] Metabolic paediatricians
- [ ] Adult metabolic doctors
- [ ] Gynaecologists/obstetricians (maternal PKU)
- [ ] Dieticians/Nutritionists
- [ ] Dieticians, metabolic diseases
- [ ] Nurses specialising in PKU
- [ ] Psychologists
- [ ] Clinical biochemists
- [ ] Research scientists
- [ ] Other (please specify)

b. Do you have a dedicated dietician to care for PKU patients at your centre?

- ○ Yes
- ○ No

If yes, to what level is your dietician trained?

- ○ General high school education only
- ○ Further education diploma (not university)
- ○ University degree
- ○ Higher degree (Masters or above)

6. Existing guidelines and protocols

a. Are you aware of published guidelines or protocols for PKU diagnosis and treatment?

- ◯ Yes
- ◯ No

If yes, which ones are you aware of (please specify)

b. Which guidelines or protocols for PKU diagnosis and treatment does your centre use? (multiple answers possible)

- ☐ Own guidelines/protocols (unpublished)
- ☐ Published guidelines/protocols

If possible, please provide details (e.g. authors, title, year)

c. Do you have a local or national PKU patient registry?

- ◯ Yes, both national and local registries
- ◯ Yes, national registries only
- ◯ Yes, local registries only
- ◯ No

d. Are there any organisations (eg. scientific societies, groups of interest, or others) dedicated to professionals involved in PKU treatment in your country?

- ◯ Yes
- ◯ No

If yes, please specify the nature of this organisation

e. Are there any organisations dedicated to PKU patients and their families in your country?

- ◯ Yes
- ◯ No

If yes, please specify the nature of this organisation

7. Service offered to patients: Phe-free protein substitutes and low-p...

a. How many kinds of Phe-free protein substitutes are available in your country?

[ ]

b. Who contributes to the costs of Phe-free protein substitutes? (multiple answers possible)

- [ ] Government
- [ ] Private health insurance
- [ ] Parents/patients
- [ ] Other (please specify)

[ ]

c. How are special dietary products delivered to PKU patients? (multiple answers possible)

- [ ] Low-protein shops
- [ ] Home delivery
- [ ] Internet
- [ ] Other (please specify)

[ ]

d. How many pharmaceutical companies supply Phe-free protein substitutes in your country? (leave blank if you do not know)

[ ]

e. How many companies supply low-protein foods in your country? (leave blank if you do not know)

[ ]

f. Who contributes to the cost of special low protein foods (flour, pasta)? (multiple answers possible)

- [ ] Government
- [ ] Private health insurance
- [ ] Parents/patients
- [ ] Other (please specify)

[ ]

g. What kind of social support is offered to PKU patients in your country? (multiple answers possible)

- [ ] Disability allowance
- [ ] Disability certificate which helps with education, employment, travel expenses
- [ ] Dietary allowance to go to summer camps
- [ ] Reimbursement of travel expenses
- [ ] Home support or compensation to parents for decreasing working hours
- [ ] No support available
- [ ] Other (please specify)

Challenges and areas for improvement in PKU care

What are the main challenges in your country in relation to screening, confirmatory diagnosis and treatment of PKU?

What would you like to be improved in your country in terms of PKU care?
