# Supplementary material for: Diagnostic and management practices for phenylketonuria in 19 countries of the South and Eastern European Region: survey results
Source: Eur J Pediatr. 2015 Sep 8;175:261–72. doi: 10.1007/s00431-015-2622-5 (PMC4724370; doi:10.1007/s00431-015-2622-5)
Supplement: Supplementary file 2 — (PDF 644 kb) [file 431_2015_2622_MOESM2_ESM.pdf]

Article title: Diagnostic and management practices for phenylketonuria in 19 countries of the South and Eastern European Region: survey results

Journal: European Journal of Pediatrics
Authors: Maria Giżewska, Anita MacDonald, Amaya Bélanger-Quintana, Alberto Burlina, Maureen Cleary, Turgay Coşkun, François Feillet, Ania C. Muntau, Friedrich Trefz, Francjan van Spronsen, Nenad Blau
Corresponding author: Maria Giżewska, Pomeranian Medical University, Szczecin, Poland, maria.gizewska@gmail.com

Online resource 2

Summary of answers to questions not presented in main results section of manuscript

Survey on PKU care in Eastern Europe and neighbouring countries

1d. With which group(s) of PKU patients are you involved? (multiple answers possible)

Answer Options	Response Percent	Response Count
All patients with PKU	75.0%	45
Only newborns, children and teenagers	11.7%	7
Only newborns, children and teenagers and PKU patients with intellectual disability	1.7%	1
Only adults and maternal PKU	1.7%	1
answered question	90.0%	54
skipped question	10.0%	6
total	100.0%	60

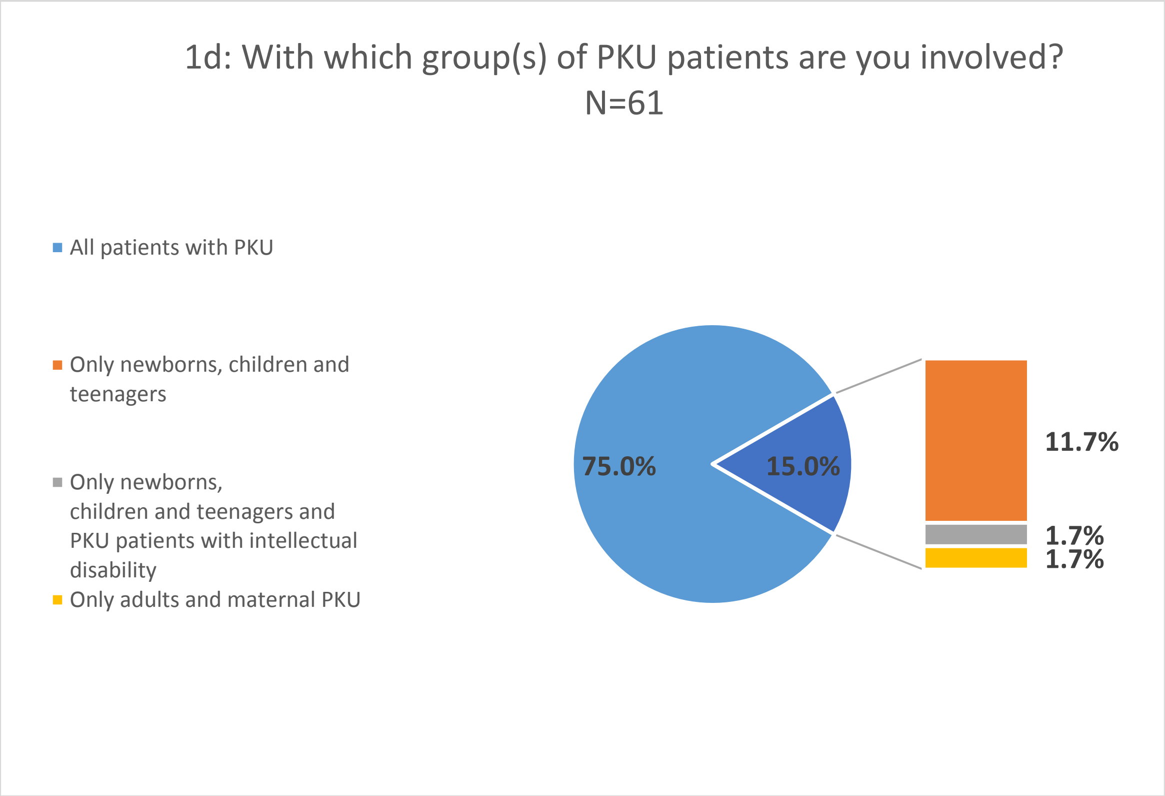

Survey on PKU care in Eastern Europe and neighbouring countries

1e. How many centres or clinics offer a PKU service in your country? (leave blank if you do not know)						
Answer Options	Total (combined answers from single country)	median	min	max	Response Percent	Response Count
Number of centres and clinics:	45	4	1	11	n/a	27
				answered question	87.1%	27
				skipped question	12.9%	4
				total	100.0%	31

Survey on PKU care in Eastern Europe and neighbouring countries

1f. Approximately how many PKU patients (treated and untreated) are there in your country? (leave blank if you do not know)						
Answer Options	Total (combined answers from single country)	median	min	max	Response Percent	Response Count
Number of patients:	15,732	350	6	10,000	n/a	21
				answered question	67.7%	21
				skipped question	32.3%	10
				total	100.0%	31

Survey on PKU care in Eastern Europe and neighbouring countries

1i. Please approximate the distribution of all PKU patients followed over the course of a typical (i.e. average) year at your clinic/centre, as follows: (leave blank if you do not know)							
Answer Options	Response Average	SD	median	min	max	Response Percent	Response Count
Percentage of children (<18 years of age):	75.4	17.8	80	40	100	87.1%	27
Percentage of adults (≥18 years of age):	24.6	17.8	20	0	60	87.1%	27
					answered question	**87.1%**	**27**
					skipped question	**12.9%**	**4**
					total	**100.0%**	**31**

Answer Options	Response Percent	Response Count
0	12.9%	4
1-10	12.9%	4
11-25	22.6%	7
26-50	32.3%	10
≥51	6.5%	2
answered question	**87.1%**	**27**
skipped question	**12.9%**	**4**
total	**100.0%**	**31**

Survey on PKU care in Eastern Europe and neighbouring countries

1m. Approximately what is the average distance (km) that PKU patients currently have to travel to your centre? (leave blank if you do not know)							
Answer Options	Response Average	SD	median	min	max	Response Percent	Response Count
Number of kilometers:	136.8	114.3	110	10	500	n/a	26
					answered question	**83.9%**	**26**
					skipped question	**16.1%**	**5**
					total	100.0%	31

Answer Options	Response Percent	Response Count
1-10	6.5%	2
11-50	19.4%	6
51-100	16.1%	5
101-200	29.0%	9
≥201	12.9%	4
answered question	**83.9%**	**26**
skipped question	**16.1%**	**5**
total	100.0%	31

Survey on PKU care in Eastern Europe and neighbouring countries

1n. Approximately how many PKU patients at your centre are followed in another country? (leave blank if you do not know)							
Answer Options	Response Average	SD	median	min	max	Response Percent	Response Count
Number of patients:	2.6	4.0	1	0	20	n/a	28
					answered question	90.3%	28
					skipped question	9.7%	3
					total	100.0%	31

Survey on PKU care in Eastern Europe and neighbouring countries

2a-ii If yes, is the programme public or private?		
Answer Options	Response Percent	Response Count
Public	96.8%	30
Private	0.0%	0
Partially public, partially private	0.0%	0
answered question	96.8%	30
skipped question	3.2%	1
total	100.0%	31

Survey on PKU care in Eastern Europe and neighbouring countries

2a-iii If yes, does it exist at a regional or national level?		
Answer Options	Response Percent	Response Count
At a regional level:	6.5%	2
At a national level:	87.1%	27
answered question	93.5%	29
skipped question	6.5%	2
total	100.0%	31

Survey on PKU care in Eastern Europe and neighbouring countries

2b. When was neonatal screening implemented? (please provide year as YYYY; eg. 2013)

Answer Options	Response Average	SD	median	min	max	Response Percent	Response Count
At a regional level:	1982	13.6	1985	1964	2009	54.8%	17
At a national level:	1989	12.5	1986	1975	2011	90.3%	28
					answered question	**96.8%**	**30**
					skipped question	**3.2%**	**1**
					total	**100.0%**	**31**

Regional	Answer Options	Response Percent	Response Count
	1960s	12.9%	4
	1970s	9.7%	3
	1980s	16.1%	5
	1990s	12.9%	4
	2000s	3.2%	1
	2010s	0.0%	0
	answered question	**54.8%**	**17**
	skipped question	**45.2%**	**14**
	total	**100.0%**	**31**

National	Answer Options	Response Percent	Response Count
	1960s	0.0%	0
	1970s	25.8%	8
	1980s	29.0%	9
	1990s	9.7%	3
	2000s	19.4%	6
	2010s	6.5%	2
	answered question	**90.3%**	**28**
	skipped question	**9.7%**	**3**
	total	**100.0%**	**31**

Survey on PKU care in Eastern Europe and neighbouring countries

2h. Who contacts parents/patients following a positive neonatal PKU screening test at your centre? (multiple answers possible)		
Answer Options	Response Percent	Response Count
Physician	9.7%	3
Paediatrician	41.9%	13
Clinical geneticist	9.7%	3
Metabolic paediatrician	29.0%	9
Dietician/Nutritionist	0.0%	0
Dietician, metabolic diseases	6.5%	2
Nurse specialising in PKU	12.9%	4
Psychologist	0.0%	0
Clinical biochemist	12.9%	4
Research scientist	3.2%	1
Other (please specify)	9.7%	3
answered question	93.5%	29
skipped question	6.5%	2
total	100.0%	31

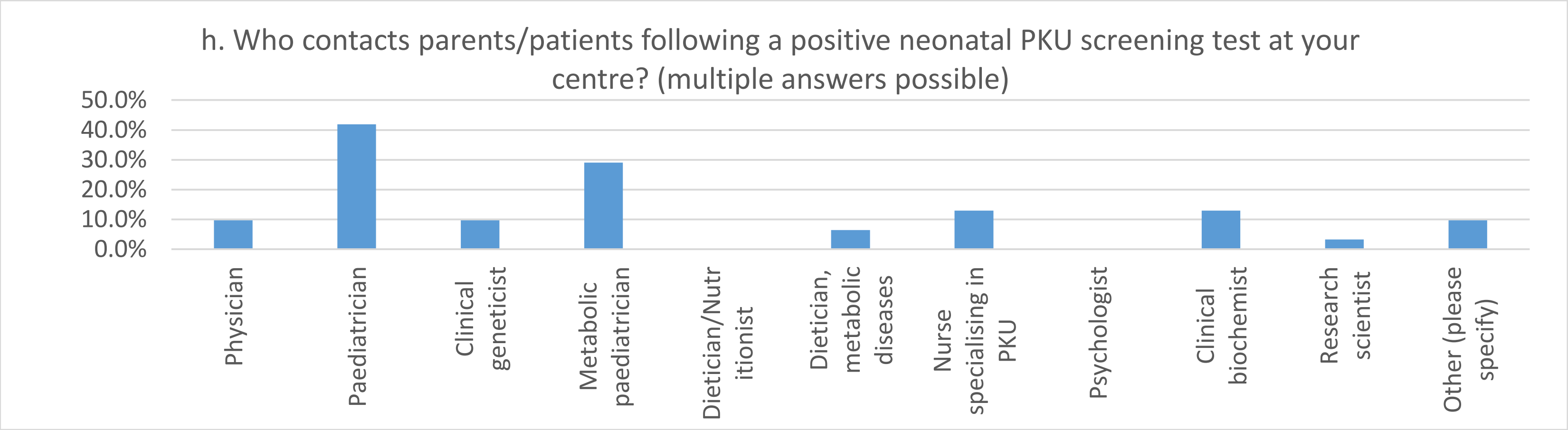

Survey on PKU care in Eastern Europe and neighbouring countries

2i. What is the method used to contact parents/patients following a positive neonatal PKU screening test at your centre? (multiple answers possible)		
Answer Options	Response Percent	Response Count
Telephone	96.8%	30
Email	6.5%	2
Other (please specify)	22.6%	7
answered question	**96.8%**	**30**
skipped question	**3.2%**	**1**
total	**100.0%**	**31**

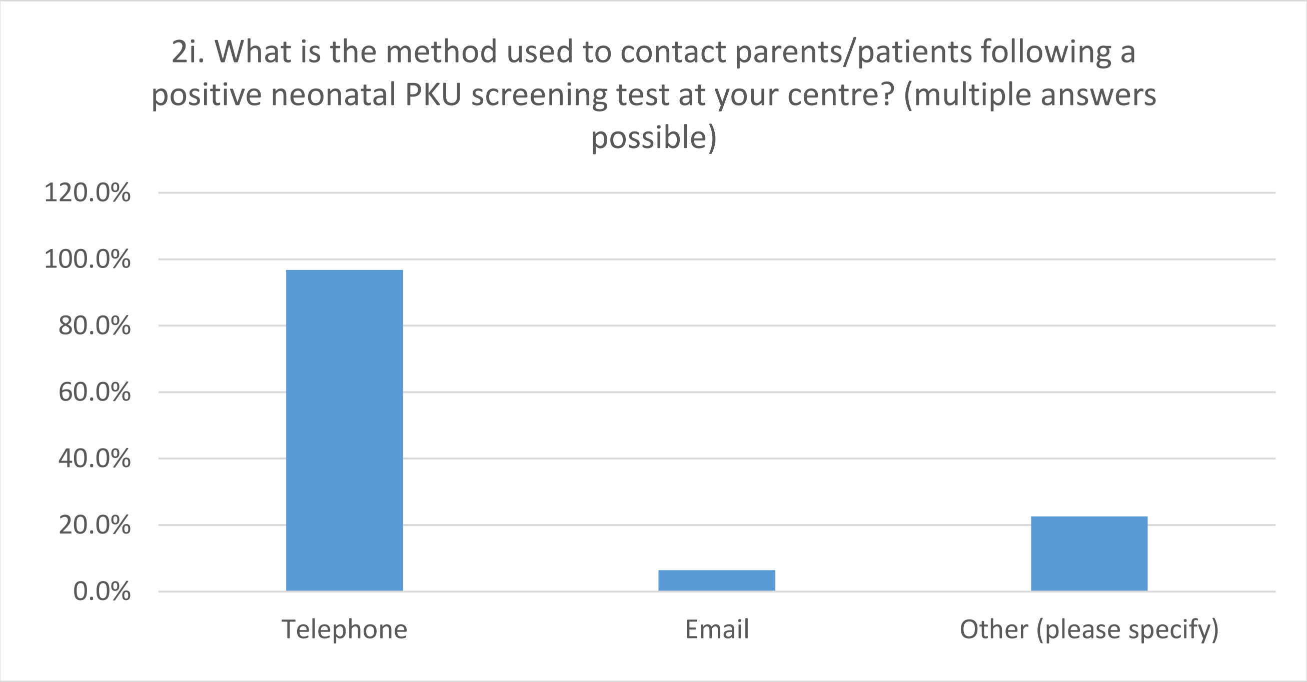

Survey on PKU care in Eastern Europe and neighbouring countries

2j. Is the confirmatory diagnosis of PKU and introduction of treatment performed for in-patients or out-patients at your centre? (multiple answers possible)		
Answer Options	Response Percent	Response Count
In-patients (i.e. patients admitted to the	71.0%	22
Out-patients (i.e. patients attending a clinic	35.5%	11
answered question	100.0%	31
skipped question	0.0%	0
total	100.0%	31

Survey on PKU care in Eastern Europe and neighbouring countries

2o. If your centre performs BH4 loading tests, are they generally done before starting with a low-phenylalanine (Phe) diet?		
Answer Options	Response Percent	Response Count
Yes	38.7%	12
No	35.5%	11
answered question	**74.2%**	**23**
skipped question	**25.8%**	**8**
total	**100.0%**	**31**

Survey on PKU care in Eastern Europe and neighbouring countries

2r-ii If yes, what is the method used for pterins analysis? (multiple answers possible)		
Answer Options	Response Percent	Response Count
in urine	32.3%	10
in dry blood spot	19.4%	6
answered question	45.2%	14
skipped question	54.8%	17
total	100.0%	31

Survey on PKU care in Eastern Europe and neighbouring countries

2t If yes, would the information be included in the BIOPKU database (www.biopku.org)?		
Answer Options	Response Percent	Response Count
Yes	19.4%	6
No	35.5%	11
I am not aware of this database	16.1%	5
answered question	71.0%	22
skipped question	29.0%	9
total	100.0%	31

Survey on PKU care in Eastern Europe and neighbouring countries

3d. Does your centre allow breastfeeding in neonates and infants with PKU?		
Answer Options	Response Percent	Response Count
Yes	96.8%	30
No	3.2%	1
answered question	100.0%	31
skipped question	0.0%	0
total	100.0%	31

Survey on PKU care in Eastern Europe and neighbouring countries

3h. BH4 treatment is:		
Answer Options	Response Percent	Response Count
Available at all PKU centres in your country	48.4%	15
Available at your centre, but not neccesarily	0.0%	0
Not available at your centre, but might be	6.5%	2
Not available anywhere in your country	38.7%	12
answered question	100.0%	31
skipped question	0.0%	0
total	100.0%	31

Survey on PKU care in Eastern Europe and neighbouring countries

3j. Do PKU patients at your centre take any of the additional supplements (besides in Phe-free protein substitutes) on the following list? (Multiple answers are possible; if the		
Answer Options	Response Percent	Response Count
Long chain polyunsaturated fatty acids	25.8%	8
Vitamins and minerals	74.2%	23
Carnitine	0.0%	0
Antioxidants	6.5%	2
Cod liver oil	22.6%	7
Other supplements (please specify)	6.5%	2
answered question	80.6%	25
skipped question	19.4%	6
total	100.0%	31

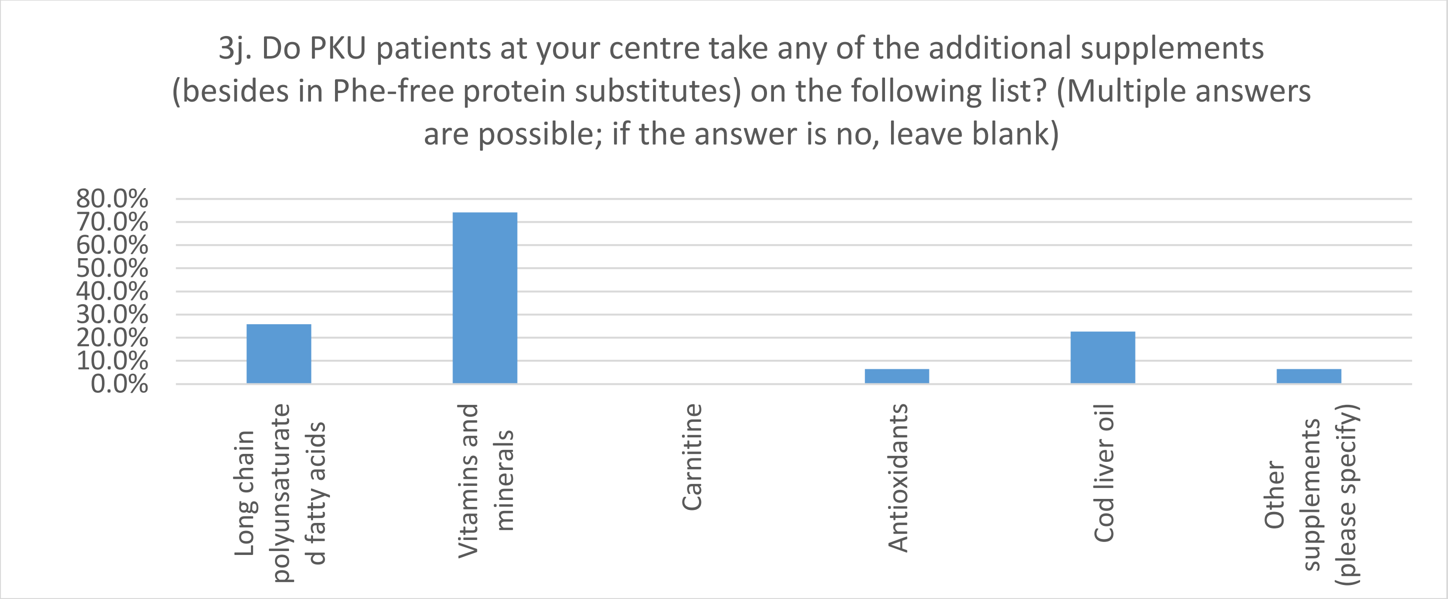

Survey on PKU care in Eastern Europe and neighbouring countries

4e. Approximately what is the average return time of routine Phe control results to your patients, after blood sampling?

Answer Options	Response Average	SD	median	min	max	Response Percent	Response Count
Number of days:	3.8	2.1	3	1	8	n/a	30
					answered question	96.8%	30
					skipped question	3.2%	1
					total	100.0%	31

Answer Options	Response Percent	Response Count
1-2	25.8%	8
3-4	35.5%	11
5-6	16.1%	5
7-8	19.4%	6
answered question	96.8%	30
skipped question	3.2%	1
total	100.0%	31

Survey on PKU care in Eastern Europe and neighbouring countries

4f. How do patients at your centre receive results of Phe levels? (multiple answers possible)		
Answer Options	Response Percent	Response Count
E-mail	41.9%	13
Phone	74.2%	23
Letter	38.7%	12
Clinic visits	54.8%	17
Other (please specify)	3.2%	1
answered question	100.0%	31
skipped question	0.0%	0
total	100.0%	31

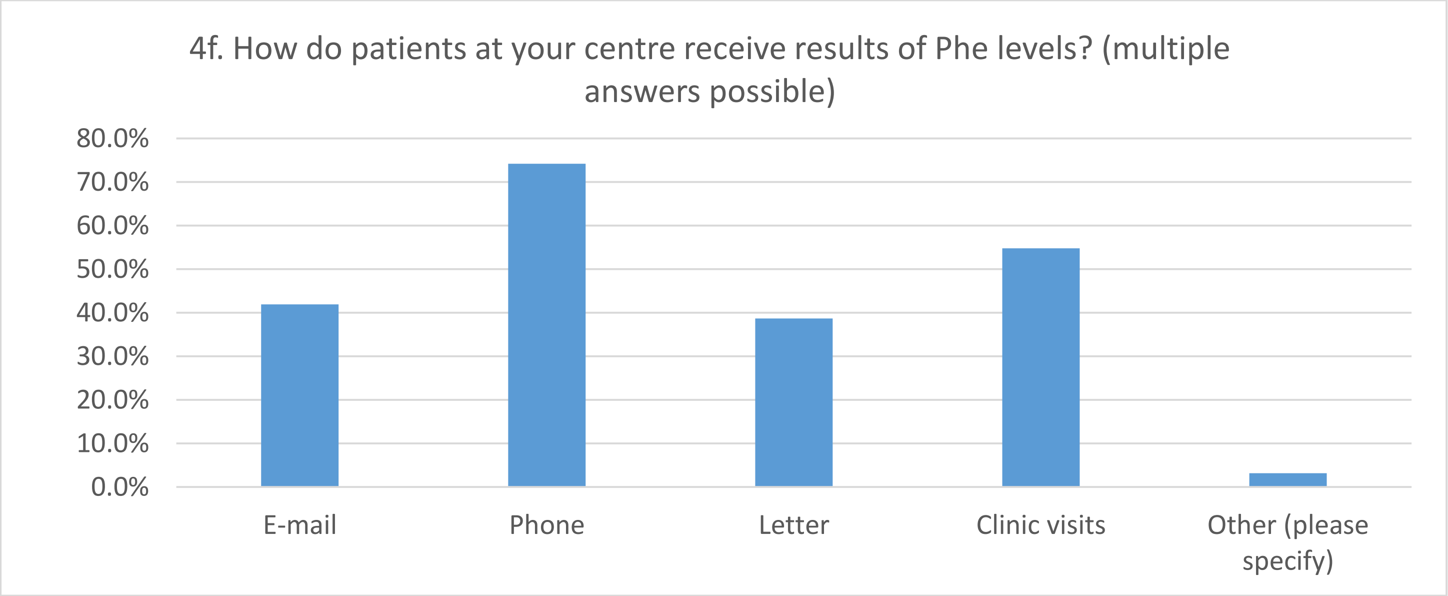

Survey on PKU care in Eastern Europe and neighbouring countries

4g. When giving the Phe results, who contacts parents/patients to provide advice or make changes to diet or other treatment? (multiple answers possible)

Answer Options	Response Percent	Response Count
Physician	0.0%	0
Paediatrician	32.3%	10
Clinical geneticist	12.9%	4
Metabolic paediatrician	54.8%	17
Adult metabolic doctor	0.0%	0
Dietician/Nutritionist	29.0%	9
Dietician, metabolic diseases	16.1%	5
Nurse specialising in PKU	0.0%	0
Psychologist	0.0%	0
Clinical biochemist	6.5%	2
Research scientist	6.5%	2
Other (please specify)	6.5%	2
answered question	100.0%	31
skipped question	0.0%	0
total	100.0%	31

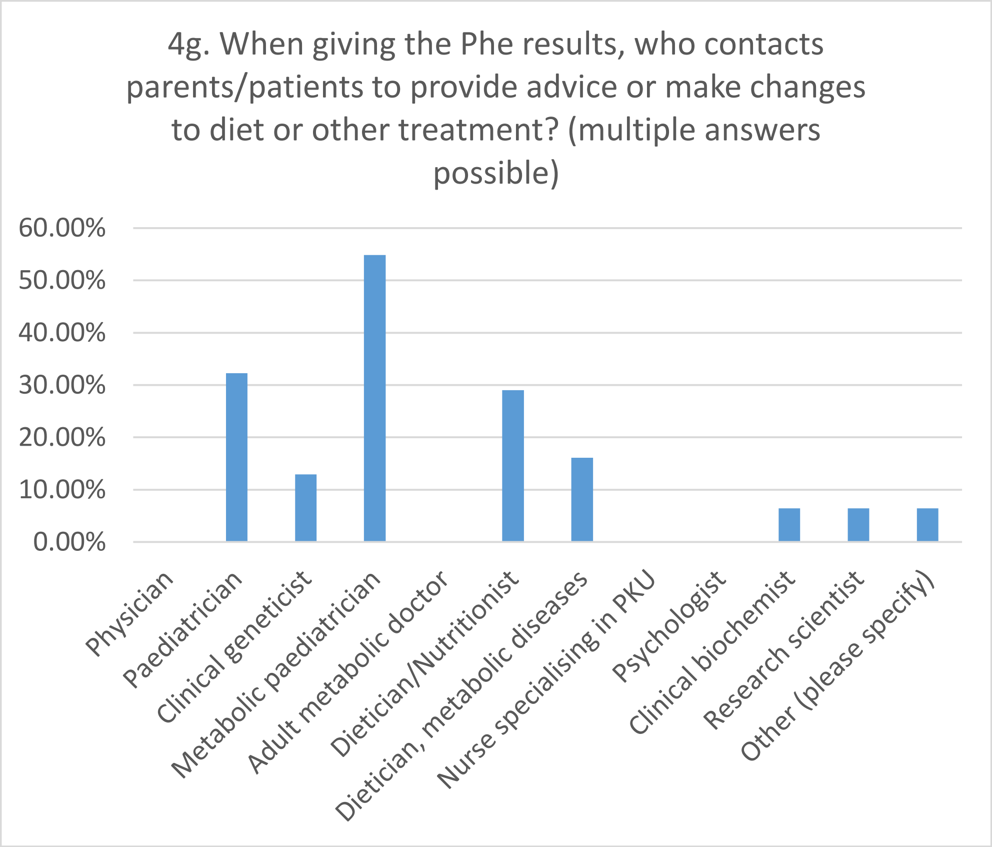

Survey on PKU care in Eastern Europe and neighbouring countries

4h. How many clinical evaluations are performed per year in children and non-pregnant adults with PKU?							
Answer Options	Response Average	SD	Median	Min	Max	Response Percent	Response Count
Newborns and infants (<1 year of age)	11.76	9.76	10	4	24	93.5%	29
Young children (1-4 years of age)	7.34	9.49	4	2	12	93.5%	29
Older children (5-10 years of age)	4.52	4.80	4	1	10	93.5%	29
Teenagers (11-17 years of age)	3.66	3.07	3	1	15	93.5%	29
Adults (≥18 years of age)	3.13	4.85	2	1	25	77.4%	24
Maternal PKU	10.45	6.39	12	1	25	64.5%	20
answered question						93.5%	29
skipped question						6.5%	2
total						100.0%	31

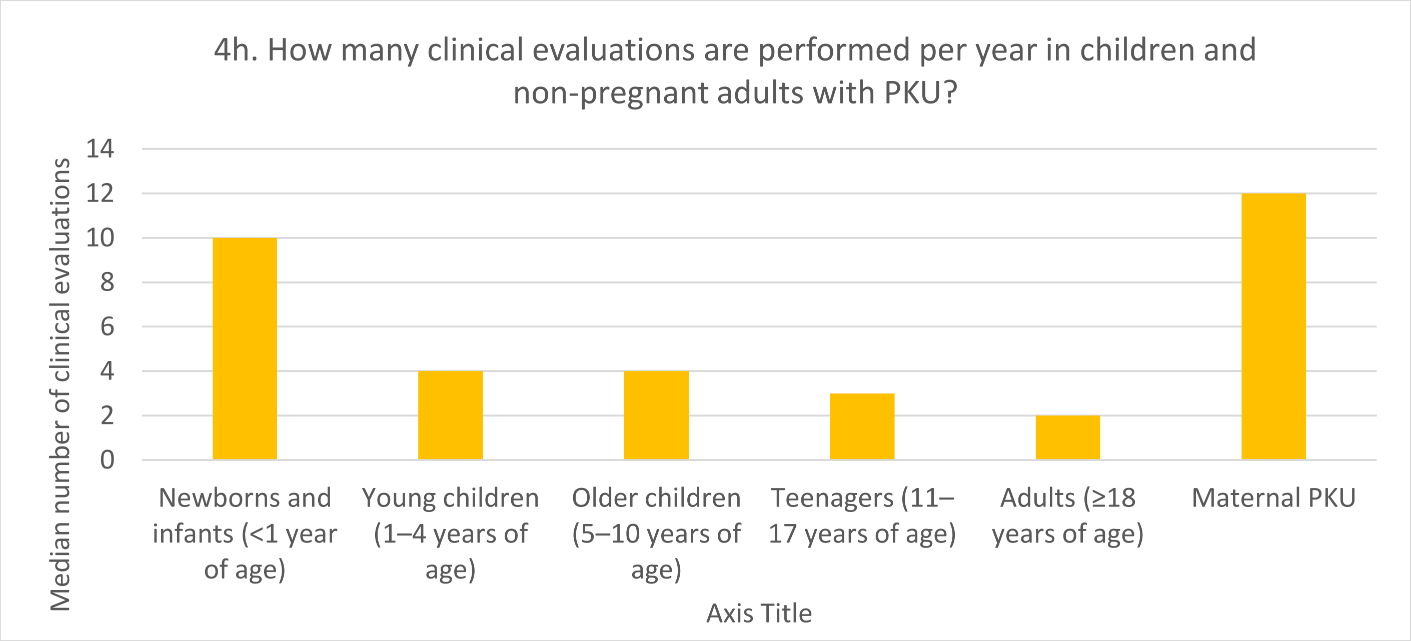

Survey on PKU care in Eastern Europe and neighbouring countries

4i. In pregnant women with PKU (maternal PKU), what is the frequency of clinical evaluations per trimester of pregnancy?						
Answer Options	Once every week	Once every two weeks	Once every month	Other (specify below)	Response Percent	Response Count
First trimester	9	5	4	2	64.5%	20
Second trimester	3	8	7	1	61.3%	19
Third trimester	3	5	11	0	61.3%	19
			answered question	*16*	67.7%	21
			skipped question	*13*	32.3%	10
			total		100.0%	31
						Question totals
				Specify here if other frequencies are used in each trimester		5

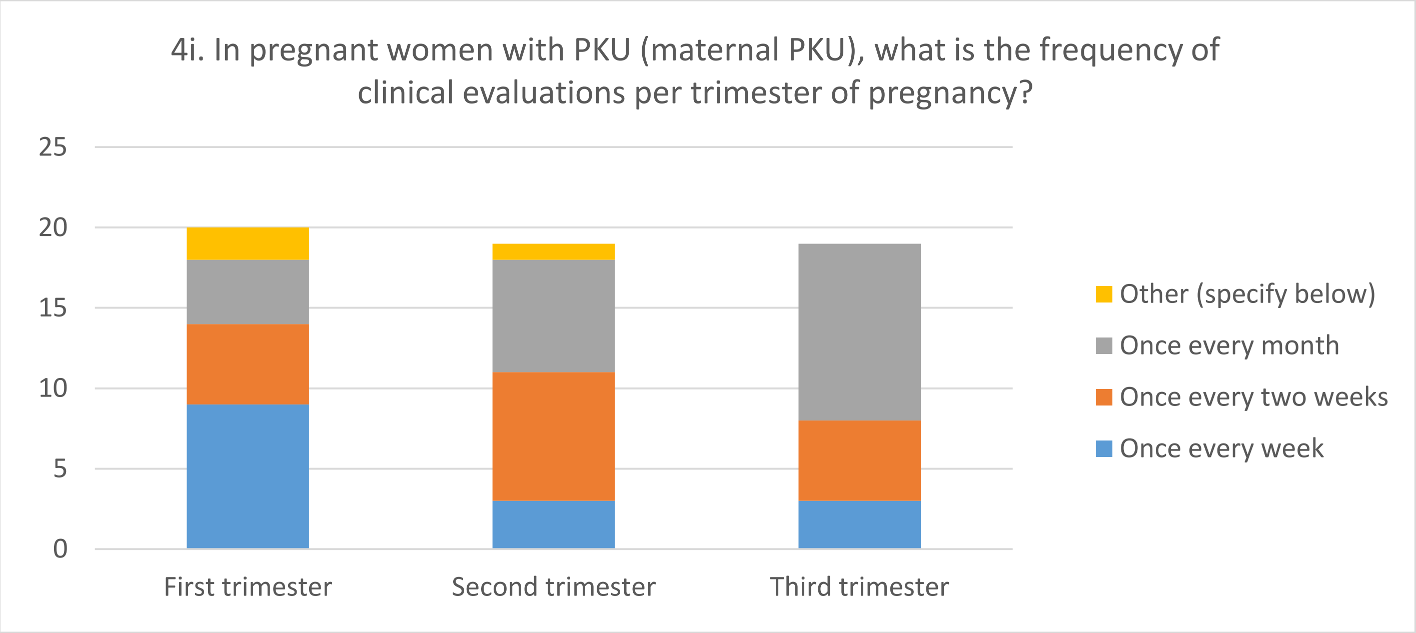

Survey on PKU care in Eastern Europe and neighbouring countries

5a. Are you aware of published guidelines or protocols for PKU diagnosis and treatment?		
Answer Options	Response Percent	Response Count
Yes	80.6%	25
No	12.9%	4
answered question	**93.5%**	**29**
skipped question	**6.5%**	**2**
total	**100.0%**	**31**

Survey on PKU care in Eastern Europe and neighbouring countries

5c. Do you have a local or national PKU patient registry?		
Answer Options	Response Percent	Response Count
Yes, both national and local registries	35.5%	11
Yes, national registries only	35.5%	11
Yes, local registries only	16.1%	5
No	12.9%	4
answered question	100.0%	31
skipped question	0.0%	0
total	100.0%	31

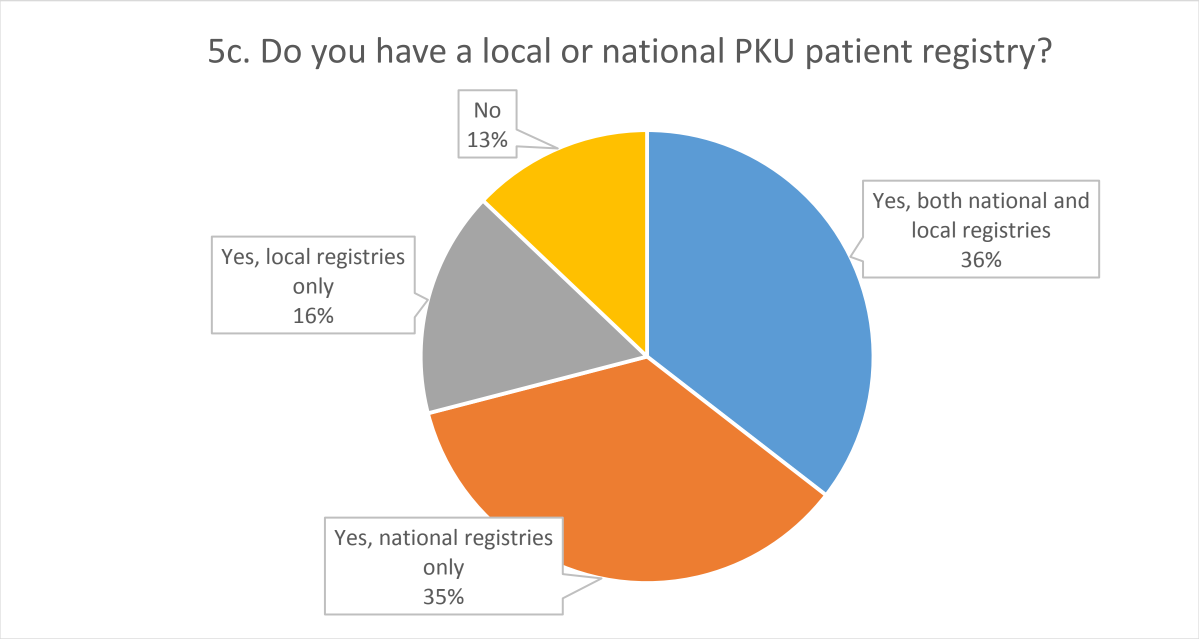

Survey on PKU care in Eastern Europe and neighbouring countries

5d. Are there any organisations (eg. scientific societies, groups of interest, or others) dedicated to professionals involved in PKU treatment in your country?		
Answer Options	Response Percent	Response Count
Yes	58.1%	18
No	41.9%	13
answered question	100.0%	31
skipped question	0.0%	0
total	100.0%	31

Survey on PKU care in Eastern Europe and neighbouring countries

6a. How many kinds of Phe-free protein substitutes are available in your country?

Answer Options	Response Average	SD	median	min	max	Response Percent	Response Count
Number of different Phe-free protein substances:	14.4	21.2	6	1	100	n/a	28
					answered question	90.3%	28
					skipped question	9.7%	3
					total	100.0%	31

Answer Options	Response Percent	Response Count
1-5	45.2%	14
6-10	19.4%	6
11-20	6.5%	2
21-100	19.4%	6
answered question	90.3%	28
skipped question	9.7%	3
total	100.0%	31

Survey on PKU care in Eastern Europe and neighbouring countries

6c. How are special dietary products delivered to PKU patients? (multiple answers possible)		
Answer Options	Response Percent	Response Count
Low-protein shops	64.5%	20
Home delivery	45.2%	14
Internet	61.3%	19
Other (please specify)	35.5%	11
answered question	100.0%	31
skipped question	0.0%	0
total	100.0%	31

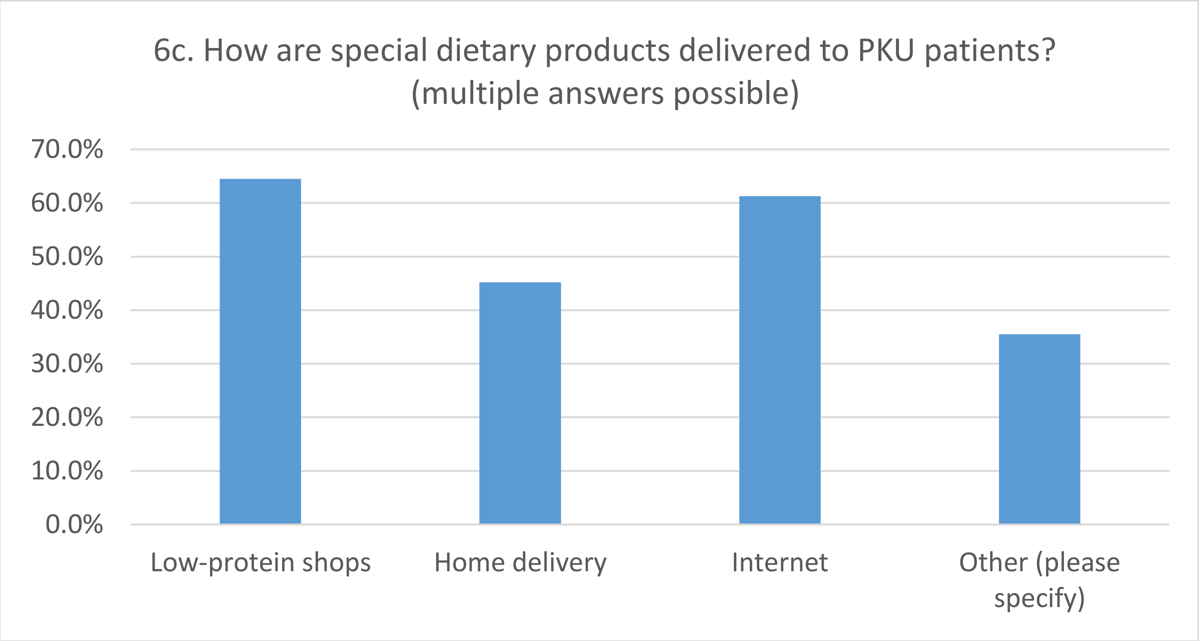

Survey on PKU care in Eastern Europe and neighbouring countries

6d. How many pharmaceutical companies supply Phe-free protein substitutes in your country? (leave blank if you do not know)							
Answer Options	Response Average	SD	median	min	max	Response Percent	Response Count
Number of pharmacentical companies:	2.6	1.0	3	0	4	n/a	26
					answered question	83.9%	26
					skipped question	16.1%	5
					total	100.0%	31

Answer Options	Response Percent	Response Count	
0	3.2%	1	
1	9.7%	3	
2	16.1%	5	
3	41.9%	13	
4	12.9%	4	
answered question		83.9%	26
skipped question		16.1%	5
total		100.0%	31

Survey on PKU care in Eastern Europe and neighbouring countries

6e. How many companies supply low-protein foods in your country? (leave blank if you do not know)							
Answer Options	Response Average	SD	median	min	max	Response Percent	Response Count
Number of companies:	4.3	4.0	4	1	20	n/a	23
					answered question	**74.2%**	**23**
					skipped question	**25.8%**	**8**
					total	100.0%	31

Answer Options	Response Percent	Response Count
1-2	25.8%	8
3-5	35.5%	11
6-10	9.7%	3
11-20	3.2%	1
answered question	**74.2%**	**23**
skipped question	**25.8%**	**8**
total	100.0%	31
