# Supplementary material for: Diagnostic and management practices for phenylketonuria in 19 countries of the South and Eastern European Region: survey results
Source: Eur J Pediatr. 2015 Sep 8;175:261–72. doi: 10.1007/s00431-015-2622-5 (PMC4724370; doi:10.1007/s00431-015-2622-5)
Supplement: Supplementary file 3 — (PDF 445 kb) [file 431_2015_2622_MOESM3_ESM.pdf]

Article title: Diagnostic and management practices for phenylketonuria in 19 countries of the South and Eastern European Region: survey results

Journal: European Journal of Pediatrics

Authors: Maria Giżewska, Anita MacDonald, Amaya Bélanger-Quintana, Alberto Burlina, Maureen Cleary, Turgay Coşkun, François Feillet, Ania C. Muntau, Friedrich Trefz, Francjan van Spronsen, Nenad Blau

Corresponding author: Maria Giżewska, Pomeranian Medical University, Szczecin, Poland, maria.gizewska@gmail.com

Online resource 3

Summary of main challenges in relation to screening, confirmatory diagnosis and treatment of PKU, and areas for improvement in PKU care (N=31)

Main challenges in screening, confirmatory diagnosis and treatment of PKU[a]	*Responses % (n)*	*Areas for improvement in PKU care*[a]	*Responses % (n)*
Lack of BH4-responsiveness testing/treatment (in all age groups)	12.9% (4)	Reimbursement for low-protein foods	16.1% (5)
Lack of funds for PKU treatment and management	9.7% (3)	Broader range of Phe-free protein substitutes/low-protein foods	16.1% (5)
Lack of TMS for diagnosis	6.5% (2)	Adequate funds for PKU treatment and management	16.1% (5)
Lack of skilled/coordinated treatment team	6.5% (2)	BH4 loading tests/treatment	12.9% (4)
		Setting up of dedicated specialist team.	12.9% (4)
		Provision of dietician	12.9% (4)
		Improved diagnostic testing (more methods/improved application)	9.7% (3)
		Newborn screening programme (Establishment/increase budget)	9.7% (3)
		Closer collaboration between medical service and external companies/organisations (pharmacies/PKU associations)	9.7% (3)
		Improved social support for patients and their families	6.5% (2)
Other[b]	25.8% (8)	Other[c]	3.2% (1)
No answer	48.4% (15)	No answer	41.9% (13)

[a]Open answers provided by participants were summarised and grouped and only those challenges/areas for improvement stated in more than 2 questionnaires are included as separate row headers in the table; [b]'Other' included: 'lack of treatment options beyond low-Phe diet', 'lack of all methods for confirmatory diagnosis', 'lack of dietician', 'lack of newborn screening', lack of PKU product availability', 'lack of reimbursement for low-protein foods', 'high cost of low-protein foods', 'lack of education'; [c]'other' included: 'Separate care for adult and paediatric patients with PKU'

BH4, sapropterin dihydrochloride; Phe, phenylalanine; PKU, phenylketonuria; TMS, tandem mass spectroscopy
